# Site‐Specific Impacts of Urbanisation on Annual Survival of a Forest Bird

**DOI:** 10.1002/ece3.71140

**Published:** 2025-05-11

**Authors:** Boglárka Bukor, Brett K. Sandercock, Karl L. Evans, Ivett Pipoly, Krisztina Sándor, András Liker, Gábor Seress

**Affiliations:** ^1^ Behavioral Ecology Research Group, Center for Natural Sciences University of Pannonia Veszprém Hungary; ^2^ Department of Terrestrial Ecology Norwegian Institute for Nature Research Trondheim Norway; ^3^ Ecology and Evolutionary Biology, School of Biosciences University of Sheffield South Yorkshire UK; ^4^ HUN‐REN‐PE Evolutionary Ecology Research Group University of Pannonia Veszprém Hungary; ^5^ HUN‐REN‐ELTE Comparative Ethology Research Group Budapest Hungary; ^6^ Balaton Uplands National Park Directorate Csopak Hungary

**Keywords:** ageing, apparent survival, breeding habitat, forest, *Parus major*, passerine, urban

## Abstract

Habitat changes associated with urbanization have major and complex effects on wildlife. In birds, urban populations often have lower reproductive success but are able to maintain similar or higher densities than non‐urban populations. One explanation proposed for this paradox is that higher survival of birds in cities may compensate for lower reproduction. We use a 9‐year dataset and Cormack‐Jolly‐Seber models to compare annual variation in apparent survival probabilities of adult great tits (Parus major) at two forests and two urban sites located in Hungary. Our analyses tested the effects of sex, age, year, population density on apparent survival, after correcting for the probability of detection. Apparent survival of great tits varied between 0.122 and 0.736, with study site and year having the greatest influence. Unexpectedly, urbanization did not have a consistent effect: the sites with the lowest and highest estimates of survival were both urban habitats. Survival probabilities at the two forest sites were similar to each other but were ~0.15 lower than survival in the best urban site and ~0.1. higher than survival in the worst urban site. Survival probabilities exhibited marked inter‐annual variation in all sites, although temporal patterns were not consistent among sites suggesting the variation was not driven by inter‐annual variation in regional scale factors. Survival probabilities decreased with bird age at both urban sites in most years, but such patterns were not detected at forest sites. Our results demonstrate that the impacts of aging on avian survival rates can diverge between urban and forest habitats, and that the demographic factors regulating urban populations can vary between locations. Age‐specific variation should be taken into account in urban ecology and further exploration of the factors driving the heterogeneity will help inform conservation of biodiversity along rural‐urban gradients.

## Graphical abstract

We used a long‐term dataset to estimate the apparent annual survival probabilities of adult great tits ( *Parus major* ) in two urban and two forest breeding habitats. Our CJS models showed the highest survival in one urban site and the lowest in the other urban site, while the two forest sites were in between the two during the study years. The age of the birds affects survival in the two urban sites, but not in the two forest sites.

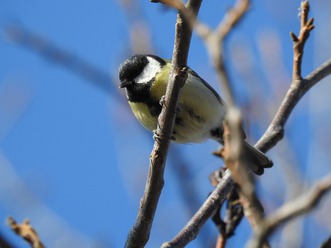

## 1 Introduction

The conversion of natural and semi‐natural habitats into human settlements is one of the key drivers of habitat loss and threats to global biodiversity (Beninde et al. 2015; Sol et al. 2017). Urban areas have grown rapidly as more people become city‐dwellers; a majority (55%) of the global human population resided in urban areas in 2018, and the proportion is projected to reach 68% by 2050 (United Nations 2018). The expansion of urban areas has complex and often negative effects on wildlife populations (Aronson et al. 2014; Ibáñez‐Álamo et al. 2016), and the impacts will likely increase in the near future.

Urban‐adapted species are commensal on human activities and reach higher densities in highly urbanised locations than taxa that require natural habitats (Blair 1996; Grade et al. 2022; Stracey and Robinson 2012). Species richness and population density are commonly used metrics to characterise the overall impact of urbanisation on local wildlife communities (Aronson et al. 2014; Batáry et al. 2018), but quantifying demographic responses provides a more direct understanding of how urbanisation affects the viability of populations (Evans 2010). A better understanding of demographic responses across the rural–urban gradient is therefore crucial if we are to preserve biodiversity in urban habitats (Banks 2004; Crawford et al. 2018). It remains unclear, however, whether populations of species that have high densities in urban settings are self‐sustaining from local reproduction or are instead maintained by immigration (Beninde et al. 2015; Chamberlain et al. 2009).

Ecological conditions can vary across the rural–urban gradient with variation in food resources, disease exposure, predation risk, and the climatic environment. Many species with high urban population densities experience reduced reproductive success in urban environments, primarily attributed to the decreased availability of natural food resources (Branston et al. 2021; Rodewald and Gehrt 2014; Vaugoyeau et al. 2016). When compared to more natural habitats, natural food resources are more limited during the breeding season for urban songbirds that rely on arthropods to feed nestlings (Seress et al. 2018; Sinkovics et al. 2021). Food limitation may also increase the workload (Jarrett et al. 2020) and hence can reduce the survival of parent birds due to investment in provisioning of nestlings. On the other hand, supplemental feeding from bird feeders, garden plants, and other anthropogenic sources may provide extra and reliable food sources that may reduce the risk of starvation and increase survival for resident birds that are capable of exploiting alternative food resources (Robb et al. 2008; Tryjanowski et al. 2018).

In addition to the consequences of food limitation, alteration of other biotic interactions can significantly impact mortality rates in urban populations, particularly concerning exposure to parasites, pathogens, and predators. Previous studies have suggested that animal populations can exhibit a higher prevalence of disease in urban settings (Bradley and Altizer 2007), where bird feeders may act as hotspots for disease transmission (but see Frątczak et al. 2021). However, urbanisation may not increase exposure to parasites, and exposure to tick‐transmitted diseases and avian malaria infections tends to be lower in urban areas (Bichet et al. 2020; Evans et al. 2009). Similarly, predation rates may also vary across the rural–urban gradient. Domestic cats can reach high densities (Loss and Marra 2017; van Heezik et al. 2010), but the diversity and density of specialist avian predators can sometimes be reduced in urban landscapes (Rodewald and Gehrt 2014; Loss et al. 2015). It is currently uncertain how the combined effect of these changes influences the overall predation risk in urban areas, especially for adult birds (Morozov 2022).

The survival probability of birds is also affected by at least two abiotic factors that vary significantly along the urban–rural continuum (Loss et al. 2015; Wilby and Perry 2006). The urban heat island effect is caused by the absorption and retention of heat by concrete buildings and infrastructure (Debbage and Shepherd 2015; Deilami et al. 2018). The resulting milder winter climate in temperate region cities relative to surrounding less urbanised landscapes may increase the overwinter survival of urban birds. On the other hand, heat island effects also amplify the intensity of summer heat waves, which have been linked to increased avian mortality (Bourne et al. 2022; Conradie et al. 2019; Pipoly et al. 2020; Sloane et al. 2022). Birds are also vulnerable to increased mortality in urban environments due to collisions with vehicles and anthropogenic structures such as building windows, power lines, and telecommunication towers (Loss et al. 2015).

Despite a large body of research that has explored how urbanisation influences particular selection pressures affecting mortality, our understanding of the cumulative impacts of the diverse drivers and thus how urbanisation shapes avian survival rates remains limited. The few studies that have been conducted reveal conflicting patterns, with some suggesting that urbanisation increases survival (Eden 2021; Horak and Lebreton 1998; Juárez et al. 2022; Phillips et al. 2018; Stracey and Robinson 2012), while others have reported negative effects (Caizergues et al. 2022; Pharr et al. 2023) or non‐linear relationships with peak survival at intermediate levels of urbanisation (Evans et al. 2015). The variation partly stems from the differences in how individual species respond to urbanisation, with notable differences even between ecologically similar species (Evans et al. 2015; Marzluff 2017). Furthermore, the environmental and ecological factors driving survival rates may significantly vary among years, but long‐term studies of urban impacts on annual survival are rare (Murgui and Hedblom 2017; Ouyang et al. 2018; Whittaker and Marzluff 2009). Accurate assessments of how survival probability changes with urbanisation also require adequate spatial and temporal replication, as highlighted in recent theoretical reviews (Streby et al. 2014; Sepp et al. 2018; Ouyang et al. 2018).

In this long‐term study, we use a temporally and spatially replicated mark‐recapture dataset for great tits *Parus major* , a small‐bodied species of woodland songbird that occupies a range of different habitats along the rural–urban gradient (Del Hoyo and Elliott 2007). Great tit population densities are, however, lower in towns than in broadleaved forests (https://www.bto.org/understanding‐birds/birdfacts/great‐tit), peak at intermediate housing densities (Tratalos et al. 2007) and are thus an urban‐adapter species (Blair 1996; Kark et al. 2007). Our objectives were to use mark‐recapture data from two urban and two forest populations of great tits in Hungary to test the impacts of urbanisation on avian survival rates and to examine whether the impacts of urbanisation are consistent among different urban environments in central Europe.

## 2 Methods

### 2.1 Study Sites

We monitored great tits breeding in nest boxes at two urban and two forest study sites in Hungary (Appendix Figure S1). One urban study site was the city of Veszprém (47°05′17.29″ N, 17°54′29.66″ E, elevation: 266 m, human population 2017: 56,927; size of the city: 126.9 km^2^; population density: 448.59/km^2^; source: https://www.nepesseg.com). We placed nest boxes in public green spaces, including university campuses, public parks, a cemetery, and the surroundings of a bus station. The nest sites are surrounded by built‐up areas and roads with heavy vehicular traffic and experience intense human disturbance. The most common native tree species are Norway maple ( *Acer platanoides* ) and European ash ( *Fraxinus excelsior* ), but other non‐native species are common, including horse chestnut ( *Aesculus hippocastanum* ) and London plane (*Platanus* sp.). A second urban study site was the town of Balatonfüred (46°57′30″ N, 17°53′34″ E, elevation: 104 m, human population in 2017: 13,888; size of the city: 46.5 km^2^, population desity: 298.66/km^2^; source: https://www.nepesseg.com). Here, nest boxes were located in a central city park (ca. 9 ha) surrounded by residential buildings and roads with heavy traffic. Human disturbance was also high at this site because the park is a popular place for jogging, dog walking, and other recreational activities, and frequently hosts cultural and community events. Small‐leaved lime ( *Tilia cordata* ), Norway maple, and sessile oak ( *Quercus petraea* ) were the most common native tree species mixed with black pine ( *Pinus nigra* ) as the dominant non‐native species. Older trees with mature canopies are abundant at both study sites, and caterpillar biomass at Balatonfüred and Veszprém was similar during the project years (Seress et al. 2018). Balatonfüred has a somewhat warmer climate than Veszprém or the two forest study sites (Nagy and Domokos 2019). The two urban study sites were located c. 15 km from each other.

The two forest study sites are located 2–3 km away from the nearest human settlements. One forest site was a mature woodland near the village of Szentgál (47°06′39″ N, 17°41′17″ E, elevation: 460 m), dominated by native European beech ( *Fagus sylvatica* ) and hornbeam ( *Carpinus betulus* ) with negligible non‐native trees. Compared to the city sites, human activity is relatively low here (Seress et al. 2021). The site is managed with selective logging and hunting in the autumn and winter. The second forest site of Vilma‐puszta was close to Veszprém (47°05′06.7″ N, 17°51′51.4″ E, elevation: 297 m) and is a protected site that is part of the Natura 2000 network. The most common trees are the native downy oak ( *Quercus cerris* ) and South European flowering ash ( *Fraxinus ornus* ) mixed with small amounts of non‐native black pine ( *Pinus nigra* ). The site has low levels of human disturbance, without paved roads, only one nearby farm, and no logging activity, but the site is occasionally visited by hikers and hunters. The two forest sites are located 15 km from each other. The Szentgál and Vilma‐puszta sites both represent common types of forests in Hungary (Borhidi 2003).

### 2.2 Field Methods

During the 9‐year study period of 2013–2021, we monitored a total of 100 to 235 great tit nesting attempts per year, depending on variation in breeding densities. Overall, between 293 and 353 nest boxes were available each year at the four study sites during the study period. The number of nest boxes varied a little over the years because a few nest boxes were removed or relocated to new places, or disappeared. Our monitoring work in Balatonfüred was completed in 2019 due to logistical constraints upon accessing the study site during years of the Covid‐19 global pandemic.

During the breeding season, from March to early July, we checked nest boxes every 3 or 4 days to monitor active nests and the development of the young. The average daily temperature (mean ± SD) during the breeding season in our study sites was: Veszprém: 15.36°C ± 1.85°C; Balatonfüred: 16.46°C ± 1.19°C; Szentgál: 13.46°C ± 1.51 C; Vilma‐puszta: 14.86°C ± 1.47°C. Breeding great tits were captured at their nests and received a unique combination of a metal ring and three plastic colour rings. Nestlings were marked with a uniquely numbered metal ring 14–16 days after hatching and were later colour‐ringed if recaptured as first‐time breeders. Parent birds were identified during the repeated nest checks and from video recordings. All nest boxes were equipped with a small, non‐transparent plastic box that conceals a small video camera. During the chick‐rearing period, the camera system enabled us to resight and reliably identify over 80% of breeding individuals each year without the need to recapture or handle birds (Seress et al. 2017). Additionally, we conducted occasional mist‐netting to increase the number of colour‐ringed birds in these populations. In our analyses, we excluded nonbreeding birds that were first captured in winter but were never observed during the breeding season. At first capture, we determined the sex and age class of tits based on their plumage characteristics and wing moult (Svensson et al. 2009). Males and females can be separated by the intensity and width of the black band on the chest and belly. We used moult limits in the coverts and flight feathers to distinguish two age classes: first‐year breeders (FY, hatched in the previous breeding season, EURING age code 5) and birds breeding after their first year (AFY, hatched at least 1 year before the previous breeding season, EURING age code 6). We refer to both age classes as adults in the rest of the manuscript. Breeding individuals regularly switched between nest boxes within all of our four study sites; however, we never detected any dispersal movements of breeding individuals moving among the four study sites in the city or forest.

### 2.3 Statistical Analysis

We estimated annual apparent survival (ϕ) and re‐sighting probabilities (*p*) of adult great tits using Cormack–Jolly–Seber models in Program MARK version 10.0 (White and Burnham 1999). We used data from colour‐ringed adult birds to create encounter histories for each individual bird. We combined ringing data with re‐sighting data from video recordings during the chick‐rearing period and coded individuals as detected (1) or not detected (0) for each year of the study. The sampling period when data on detections of birds in the populations were collected corresponded to the 4‐month breeding season, with annual survival including overwinter survival to the next year.

We analysed the live encounter data using two sets of CJS models. In the first set of models (‘combined models’), we analysed the four study sites together. In the second set of analyses (‘site‐specific models’), we modelled apparent survival separately for each of the four study sites. Using this combined approach, our two goals were: (1) to determine if there were any site effects on apparent survival; and (2) to obtain a more detailed picture of how sex, age and other factors affected apparent survival at each of the four sites.

In the combined models, we modelled apparent survival from the four study sites in the 7‐year period between 2013 and 2019 when all sites were monitored simultaneously. We captured and marked a total of 907 adult tits during this study period; 340 (37.5%) of the birds were re‐sighted or recaptured in at least one subsequent year. In the combined model set, we tested if apparent survival (ϕ) and re‐sighting probability (*p*) were (i) constant, (ii) site‐dependent among Veszprém (city), Balatonfüred (city), Szentgál (forest), or Vilma‐puszta (forest), (iii) time‐dependent (with sequential time‐periods defined as the period spanning from one breeding season to the next), (iv) sex‐dependent (male or female), or (v) dependent on the site‐specific breeding density (calculated separately for each year and site from the number of breeding pairs using nest boxes per the size of the study site (km^2^). The number of nest boxes per site per year available for great tits varied from 36 to 110 at the four sites (Appendix Table S1). We also created several combinations of these five main effects with and without interactions, generating a total of 62 candidate models (see complete model list in the Appendix Table S2). In our analysis, the goal was to assess the effect of study site on apparent survival whilst taking into account variation among years, and sex and density‐dependent effects. We were unable to include the relative age of birds in the combined models because the inclusion of age dependence would result in model overfitting due to a high number of parameters resulting from the high number of factor‐level combinations.

In the site‐specific models for each of the four sites, we modelled apparent survival for an extended 9‐year period between 2013 and 2021 at Veszprém, Szentgál, and Vilma‐puszta, and between 2013 and 2019 at Balatonfüred, thereby maximising the number of observations available from the sites where the study continued after 2019. Evaluating sites separately allowed the inclusion of age‐class in the analyses because a lower number of parameters had to be estimated for the site‐specific than the combined models, thereby reducing problems arising from model overfitting. The number of birds, and the number of birds that were re‐sighted in at least one subsequent year, were as follows: Veszprém (urban): *n* = 354 (175, 49.4%); Balatonfüred (urban): *n* = 153 (46, 30%); Szentgál (forest): *n* = 346 (138, 49.9%); Vilma‐puszta (forest): *n* = 186 (69, 37.1%). In the site‐specific analyses, we evaluated alternative models where apparent survival (ϕ) might be (i) constant, (ii) age‐dependent, (iii) time period‐dependent, or (iv) sex‐dependent (or any combination of these effects). In the age‐dependent model, the apparent survival of FY individuals after first marking (ϕ1) was treated separately from later transitions. The second age‐class included the subsequent years after FY birds had matured into the AFY age class, which were pooled with apparent survival rates of birds first marked as AFY individuals (ϕ2). If survival or site fidelity improve with age and experience, we expected that AFY birds would have higher apparent survival than FY birds. We considered candidate models where re‐sighting probability (*p*) was (i) constant, (ii) time‐dependent, or (iii) sex‐dependent (or any combination of these effects). In this set of analyses, we constructed 56 models in total for each site (see the model list in the Appendix Tables S3–S6). Parameter counts differed among sites because a shorter time series was available for Balatonfüred.

We tested for overdispersion in our data as a first step for each analysis. We fit a global model that allowed apparent survival to vary as a function of site/age category, sex, and time‐period (plus the breeding density in the combined model part) and the re‐sighting probability to vary as a function of sex and time. We tested the fit of the global model for overdispersion in the data by estimating the variance‐inflation factor (ĉ) using Fletcher's c‐hat procedure (Cooch and White 2022) in Program MARK (Fletcher's ĉ = 0.943). We compared candidate models using the Akaike information criterion corrected for small sample sizes (AICc). In the special case where the number of parameters in two models differed by one, we inspected confidence intervals for the slope coefficient to confirm that the extra parameter was informative (Arnold 2010). We also conducted model averaging across all models to obtain overall weighted estimates for apparent survival (ϕ) and used model averaged values to illustrate our results.

## 3 Results

### 3.1 Combined Models for all Four Study Sites

**FIGURE 1:**
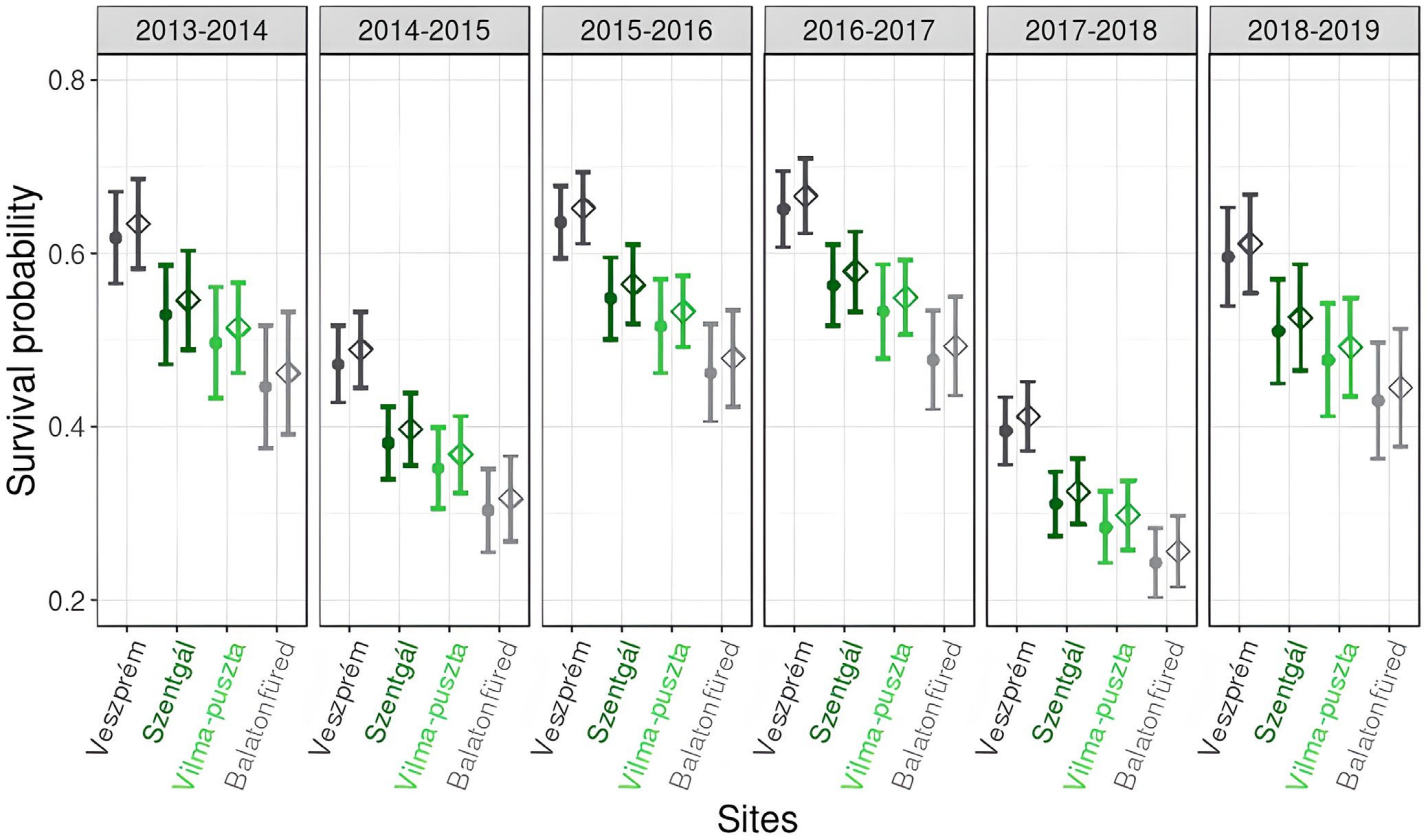
Model‐averaged estimates from the combined models of annual apparent survival estimates (φ) for adult great tits in two urban (Veszprém, Balatonfüred; grey colors) and two forest sites (Szentgál, Vilmapuszta; green colors) between 2013 and 2019. Sites are ordered from highest to lowest survival estimates. The points and squares represent estimates of apparent annual survival (φ) and the whiskers represent standard error. The filled points show the survival probabilities for females, and the empty squares show the survival probabilities for males.

The combined analyses included all four study sites, and three models were supported where two models had ∆AICc values < 2 relative to the one model with the lowest AICc value (Appendix Table S2). Effects of study site and year on apparent survival were supported by all three of these models (Table 1). The effect of sex was supported by one model with slightly higher apparent survival estimates for males than females, whereas none of these models supported an effect of breeding density on apparent survival (Table 1). The two urban study sites showed the greatest difference, with great tits having the highest apparent survival in Veszprém but the lowest in Balatonfüred (Figure 1). The probability of apparent survival at the two forest sites was intermediate to the two urban sites in all years (Figure 1). We also found evidence for large fluctuations in apparent survival among years. From 2017 to 2018, the best‐fit models estimated an apparent survival probability of ~0.30, which is half of the values estimated for the period with the highest apparent survival, 2016–2017. The probability of resighting was either constant (for model 1 and model 2: *p* = 0.814 ± 0.025) or slightly higher in females than males (model 3: females, *p* = 0.820 ± 0.035; males, *p* = 0.808 ± 0.033; Table 1).

**TABLE 1:** Supported models from the combined set of models resulting from model selection of Cormack–Jolly–Seber capture‐recapture analyses of data on breeding populations of adult great tits, at two urban sites (Veszprém, Balatonfüred) and two forest sites (Szentgál, Vilma‐puszta) between 2013 and 2019 (φ: Apparent, annual survival probability; p: Re‐sightABLEing probability).

Model	AICc	∆AICc	AICc weight	Model likelihood	Nr. of parameters	Deviance	Fletcher's ĉ
1) φ (site + time), p (.)	1874.516	0.000	0.331	1.000	10	294.045	1.049
2) φ (site + sex + time), p (.)	1875.113	0.596	0.240	0.742	11	292.604	1.044
3) φ (site + time), p (sex)	1876.490	1.973	0.123	0.372	11	293.981	1.048

*Note:* The supported models (ΔAICc < 2) from the 62 Cormack–Jolly–Seber models containing the possible combinations of parameters are shown in order of AICc. Statistics given for each model include the Akaike information criterion corrected for small sample size (AICc), relative support for each model (the AICc weight), number of parameters, likelihood and deviance (deviance: −2logL(θ) for a given model with parameters θ). A complete list of considered Cormack–Jolly–Seber capture‐recapture models is reported in Appendix Table S2.

### 3.2 Site‐Specific Models

#### 3.2.1 Veszprém (Urban)

**FIGURE 2:**
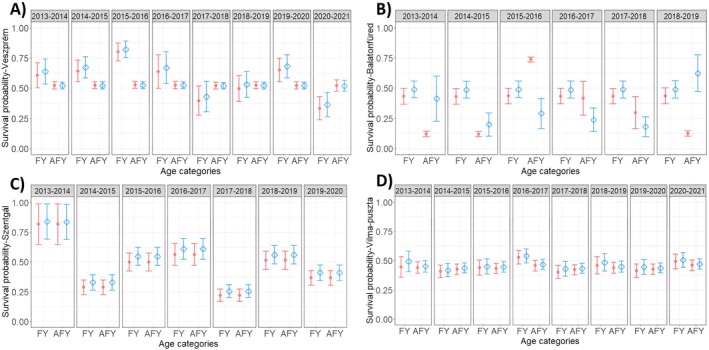
Model‐averaged estimates from the site‐specific models of annual apparent survival estimates (φ) for adult great tits in (A) Veszprém (city), (B) Balatonfüred (city), (C) Szentgál (forest) and (D) Vilma‐puszta (forest). The last transition from Szentgál is removed from the figure (because both the φ and p have time dependence, and the last transition is nonidentifiable here). The symbols indicate the estimates of φ: the red filled points represent female survival and the blue hollow squares represent male survival; whiskers represent standard error. FY: first‐year breeder = hatched in the previous calendar year (φ 1); AFY: having at least one breeding season before the current one = hatched at least two years prior (φ2).

Three models received similar support and had ∆AICc < 2 (Appendix Table S3). All three models showed that apparent survival differs between the FY and AFY age categories. The survival of FY individuals (φ1) varied among years and was unexpectedly higher than the apparent survival of AFY birds (φ2), which was constant throughout the study period (Figure 2A and Table 2). One of the top models also showed sex dependence in apparent survival, indicating a slightly better apparent survival for males. The re‐sighting probability was either constant (for model 1 and model 2: *p* = 0.888 ± 0.025) or slightly sex‐dependent (model 3: males, *p* = 0.905 ± 0.032; females, *p* = 0.869 ± 0.039).

**TABLE 2:** Supported models resulting from model selection of Cormack–Jolly–Seber capture‐recapture analyses for data on breeding populations of adult great tits at our four study sites: Veszprém (city), Balatonfüred (city), Szentgál (forest) and Vilma‐puszta (forest).

Study site	Model	AICc	∆AICc	AICc weight	Model likelihood	No. of parameters	Deviance	Fletcher's ĉ
Veszprém (urban)	(1) φ1 (time), φ2 (.); p (.)	935.029	0.000	0.278	1.000	10	173.368	0.960
	(2) φ1 (sex+time), φ2 (.); p (.)	935.768	0.738	0.192	0.691	11	172.029	0.959
	(3) φ1 (time), φ2 (.); p (sex)	936.596	1.566	0.127	0.456	11	172.858	0.949
Balatonfüred (urban)	(1) φ1 (.), φ2 (sex*time); p (.)	242.270	0.000	0.467	1.000	10	51.477	0.995
	(2) φ1 (sex), φ2 (.); p (time)	244.047	1.776	0.192	0.441	6	62.095	0.980
Szentgál (forest)	(1) φ (sex + time); p (time)	754.027	0.000	0.265	1.000	15	144.034	1.000
	(2) φ (time); p (time)	754.351	0.323	0.225	0.850	14	146.486	1.002
	(3) φ (sex + time); p (sex + time)	754.682	0.654	0.191	0.720	17	140.405	0.996
Vilma‐puszta (forest)	(1) φ (.); p (sex+time)	391.352	0.000	0.250	1.000	7	126.029	1.011
	(2) φ (.); p (time)	391.780	0.428	0.201	0.803	6	128.571	1.026
	(3) φ (sex); p (sex + time)	393.293	1.941	0.094	0.378	8	125.839	1.012

*Note:* Parameters included φ: Apparent, annual survival probability; p: Re‐sighting probability. The supported models (ΔAICc < 2) from the 56 Cormack–Jolly–Seber models containing the possible combinations of parameters are shown in order of AICc. Statistics given for each model include the Akaike information criterion corrected for small sample size (AICc), relative support for each model (the AICc weight), number of parameters, likelihood and deviance (deviance: −2logL(θ) for a given model with parameters θ). The complete list of considered Cormack–Jolly–Seber capture‐recapture models is reported in Appendix Tables S3–S6. The apparent annual survival of FY individuals in their first year (φ 1) was considered to differ from birds first marked as AFY individuals combined with subsequent years when FY birds matured into the AFY age class (φ 2).

### 3.3 Balatonfüred (Urban)

Two competing models had ∆AICc < 2 (Appendix Table S4). Apparent survival differed between the FY and AFY age categories in both models, with FY birds again tending to have higher survival than AFY birds in most years (Figure 2B). Model 1 indicated constant apparent survival for φ1 with time and sex effects on φ2 (slightly higher apparent survival estimates for males than females; Figure 2B and Table 2). However, model 2 showed sex‐dependent apparent survival for φ1 and constant apparent survival for φ2 (Table 2). The re‐sighting probability was constant in the first model (*p* = 0.837 ± 0.084), but time‐dependent in the second model (Table 2).

#### 3.3.1 Szentgál (Forest)

Three competing models had similar support with ∆AICc < 2 (Appendix Table S5). Unlike the two urban sites, the best‐fit models did not include an age effect on apparent survival (Table 2). Two out of three models indicated a weak effect of sex, with slightly higher apparent survival estimates for males than females. All three models included evidence for inter‐annual variation in apparent survival (Figure 2C). Re‐sighting probability was time‐dependent in one model and time‐and slightly sex‐dependent in the other two models (Table 2).

#### 3.3.2 Vilma‐Puszta (Forest)

Three competing models had ∆AICc < 2 (Appendix Table S6). Similar to the other forest site, the top models did not include an age effect on apparent survival (Table 2). Two out of the three supported models show constant apparent survival during the study period, whereas the third one indicated a sex effect with slightly higher apparent survival probability for males than females (Figure 2D and Table 2). The re‐sighting probability was time‐dependent in all three models, with two of these also having a sex effect (Table 2).

## 4 Discussion

We used live encounter data of breeding birds attending nests to estimate apparent annual survival of adult great tits at two urban and two rural sites across multiple years. Our long‐term field study and analyses yielded three major findings. First, we did not find consistent differences in the apparent survival estimates of great tits between urban and forest sites. Instead, both the set of combined models with all four study sites together and the site‐specific models confirmed that urban sites had both the highest (Veszprém) and the lowest (Balatonfüred) estimates of apparent survival. Specifically, in Veszprém, the average annual survival was roughly ~0.57, while in Balatonfüred it was ~0.4. The two forest sites had intermediate annual apparent survival compared to the two cities (with ~0.47 in Szentgál and ~0.45 in Vilma‐puszta). The apparent survival of great tits in Hungary was similar to previous estimates reported for populations in France, where the urban birds survival was 0.44–0.45, and in the forest site it was 0.41–0.62 (Caizergues et al. 2022), and somewhat higher than in Estonian populations, where the average annual survival ranged between 0.34 and 0.47 in urban sites and 0.26–0.38 in rural sites (Horak and Lebreton 1998). Our finding illustrates the heterogeneity of urban areas, contrasting with the prevailing viewpoint that towns and cities are homogeneous environments (Bartos Smith et al. 2016). Moreover, the site differences clearly demonstrate a need for urban ecology research to assess patterns at multiple sites (Kinnunen et al. 2022). Second, our results also highlighted considerable inter‐annual variation in apparent survival probabilities in our study populations. Variation in demographic rates underscores the importance of assessing urbanisation impacts over multiple years. Last, our site‐specific analyses also revealed evidence for reduced survival rates among older birds (FY versus AFY) in urban environments but not in forests. Survival rates typically improve with age, so unexpected declines suggest an unexpected mechanism may be acting on great tits. Declines are compatible with theoretical models of how urbanisation influences actuarial senescence (Watson et al. 2015) although empirical tests on long‐lived raptors have failed to support this hypothesis (Sumasgutner et al. 2019). In combination, our findings suggest that the processes determining apparent survival probabilities vary across urban and more natural habitats in a complex manner.

### 4.1 Limitations of the Study

Like all studies using mark‐recapture methods in open populations, it is difficult to distinguish losses to mortality from permanent emigration from the study areas. To mitigate the potential biases stemming from natal dispersal, we excluded hatch‐year birds from our dataset because natal dispersal of juveniles away from their site of birth to their first breeding site is quite common in songbirds (Könczey et al. 1997; Whittaker and Marzluff 2013). On the contrary, breeding adults typically exhibit strong site fidelity and reoccupy their territory from the previous years' range (Harvey et al. 1979). Great tit breeding dispersal typically only occurs over small distances (Harvey et al. 1979), and we consider that such events are unlikely to bias our conclusions regarding between‐site variation in survival rates for several reasons. First, our combined models did not indicate a site‐effect in the birds' re‐sighting probability, and we had no evidence for site differences in breeding propensity. Second, our previous results showed no significant difference in the nest box fidelity of breeding females between years across the four study sites (Bukor 2017). Third, due to habitat fragmentation, the urban study site at Veszprém comprises three separate habitat patches (the sizes of the patches 0.216, 0.06 and 0.055 km^2^; the two smaller patch were ~600 and ~450 m from the larger patch and ~1250 m apart as the crow flies), contrasting with the single and more evenly shaped urban park in Balatonfüred, with an area size of 0.159 km^2^ (Appendix Figure S1). Therefore, given the differences in site configuration, a dispersing bird is more likely to permanently emigrate from Veszprém than from the Balatonfüred study site. Nevertheless, we found higher apparent survival rates at Veszprém, suggesting that the differing estimates of apparent annual survival for the two cities are not unduly influenced by emigration from our study area. Last, both survival and site fidelity are expected to increase with age, but apparent survival actually decreased among older tits at urban but not forest sites.

Our combined models indicated that the birds' re‐sighting probability was constant in two of three supported models, while slightly higher for females in the third one (*p* > 0.8 in all of these models). A marginal difference in detectability between sexes is probably explained by our data collection protocol. Specifically, during nest checks, the likelihood of finding one of the parent birds on the nest—and thus identifying it by its colour rings—is likely to be higher for females because they are the sex that is more likely to remain on the nest during the incubation or brooding period (Vincze et al. 2021). Additionally, when capturing birds at their nest, we preferentially targeted females over males in situations where we had to choose which parent to capture because we focused on females for some of our other study objectives. Nevertheless, the overall high re‐sighting probabilities are consistent with our intensive nest monitoring programme and the use of the camera system for identifying individual birds, confirming that we could successfully identify the majority of adults breeding in the nest boxes.

### 4.2 Differences Among Sites and Habitats

Our results provided no evidence that survival rates of songbirds were consistently higher in urban environments, which was in contrast with previous studies in other passerine species (Evans et al. 2015; Juárez et al. 2022). Several factors may explain higher spatial heterogeneity among urban sites. First, the availability of insect larvae and other important natural food sources is markedly lower in cities compared to forests, with less pronounced peaks in abundance throughout the breeding season (Marciniak et al. 2007; Nadolski et al. 2021; Seress et al. 2018). Adult birds may compensate for lower availability and less predictable natural food sources in urban areas by exploiting anthropogenic food sources (Sinkovics et al. 2021, 2023). However, the availability of alternative food sources, such as bird feeders, trash bins, and pet or domestic animal food, depends on local conditions and is likely to vary widely between urban areas (Tryjanowski et al. 2015). For instance, in the winter of 2021–2022, the mean density of active bird feeders in the Veszprém study site was 126.3 feeders/km^2^ (Bukor et al. 2024). We do not have comparable data for Balatonfüred, but supplemental feeding is likely to be lower since most of the site was a summer holiday area characterised by parkland with a lower density of people and fewer inhabited houses. Therefore, while some urban locations may offer abundant anthropogenic food sources potentially increasing the annual survival of birds compared to natural habitats (Sepp et al. 2018), supplementary resources may be scarcer in other urban areas. In the latter case, birds may either maintain lower body reserves with less fat, increasing the risk of starvation under adverse weather conditions, or may be forced to move more searching for food, increasing the risk of predation‐related mortality (Baker et al. 2005; Loss et al. 2015; van Heezik et al. 2010).

Second, cities may also differ in predator densities, which could have a strong impact on adult survival rates. Several raptors known for hunting small birds, such as the Eurasian sparrowhawk ( *Accipiter nisus* ), the Eurasian goshawk ( *A. gentilis* ), and the common kestrel ( *Falco tinnunculus* ) have successfully colonised European cities (De Merling Chapa et al. 2020; Millsap 2018; Thornton et al. 2017). Additionally, domestic predators like feral cats can be abundant in urban areas (Loss et al. 2015; Loss and Marra 2017) posing a significant threat to adult birds (Baker et al. 2005; Bonnington et al. 2013). Since the characteristics of local predator communities could differ between our study sites, predation pressure may also be involved in generating the heterogeneity of apparent survival rates we found between the two cities involved in our study.

Third, microclimatic conditions can also influence the survival rate differences among urban bird populations. For example, the urban heat island effect, which is more pronounced in larger cities (Climate Central 2021), acts as a buffer against the effects of cold weather conditions, potentially enhancing winter survival in more urbanised areas. Previous research has found that milder winters contribute to higher adult survival rates in several passerine species (Dybala et al. 2013; Salewski et al. 2013). However, differences in microclimate are probably not applicable to our study sites, because Balatonfüred had the lowest apparent survival estimates but was actually the warmest site among our study locations. On the other hand, urban heat island effects can intensify the effects of extreme temperature events, such as summer heat waves, thereby increasing heat stress in more urbanised areas (Pipoly et al. 2020; Xie et al. 2017). Previous studies have shown that the escalation of summer heat waves can increase adult mortality rates in both desert and temperate species (Gardner et al. 2017; McKechnie et al. 2021; Lv et al. 2023), but the degree of impact varies significantly among species (Robinson et al. 2007; Gullett et al. 2014). According to this finding, the increased mortality due to heat stress in Balatonfüred, relative to the other study sites, could partially account for the consistently lowest survival rates we found at this site (Figure 1).

### 4.3 Differences Between Age Groups

Our site‐specific models revealed that age class significantly influenced apparent survival at both urban sites, but the pattern was absent in the forest sites. Unexpectedly, FY (younger) birds had consistently higher apparent survival than AFY (older) birds in urban habitats. One explanation could be that urban stressors (e.g., exposure to toxins) might accelerate aging and their additive effects could reduce the survival chances of older age classes. Indeed, several studies confirmed the bioaccumulation of various metallic trace elements in parid species breeding in industrial or highly urbanised areas (Chatelain et al. 2021; Costa et al. 2013).

An alternative explanation for the difference between age classes is that it could indicate a higher cost of reproduction in urban environments if older birds have greater investment in reproduction than first‐time breeders. Previous research indicates that increased parental investment can detrimentally affect subsequent survival. For example, the survival rate of female great tits increased when their seasonally second clutches were experimentally removed (Verhulst 1998). The cost of reproduction hypothesis may be particularly relevant for great tits, as studies have found that the survival rate of female willow tits ( *Poecile montanus* ) declined significantly after reaching 5 years of age, while such an effect was not present in males (Orell and Belda 2002). Recent studies also revealed that great tits experienced a reduction in telomere length between winter and the subsequent reproductive season in the urban but not in the forest habitat (Saulnier et al. 2022), despite urban birds' generally better tolerance for stressful conditions (Costantini et al. 2014; Walthers and Barber 2020). The findings suggest that the demographic costs associated with breeding may emerge in later life stages, highlighting the intricate balance between reproductive investment and survival in birds that might also be affected by breeding habitat type.

Similar to our results, a study on four blue tit populations in the Mediterranean region found a consistently higher survival for first‐year versus older birds in forested areas (Bastianelli et al. 2021). In contrast, a recent study in France reported no significant difference between first‐year and adult great tits' survival probabilities at an urban site but reported the highest survival for yearling birds in forest habitats (Caizergues et al. 2022). The comparison of these two studies to ours is not straightforward, however, due to the different age‐categorisation applied, and because in previous studies yearling birds' survival estimates might have been confounded by natal dispersal among yearling birds.

### 4.4 Differences Between the Sexes

An effect of sex was supported in some of the models for each study site, and also in our combined models, indicating slightly higher apparent survival for males than females both in our forest and urban sites. Our findings are consistent with several previous studies on great tits (Clobert et al. 1988; Forero et al. 2001; Masoero et al. 2016; Callery et al. 2022;), although some mark‐recapture studies reported no significant difference between sexes (Caizergues et al. 2022), or even slightly higher apparent survival in females (Horak and Lebreton 1998).

In birds, the adult sex ratio tends to be biased towards the homogametic sex (males) due to the higher mortality rate of the heterogametic sex (females; Pipoly et al. 2015; Székely et al. 2014). Sex differences are often explained by the higher reproductive costs faced by females, including egg‐laying and more intensive brood care, which could reduce their survival probability (Romano et al. 2022; Verhulst 1998). Despite a general pattern of sex differences, our results suggesting higher reproductive costs for females relative to males in both habitat types were unexpected. Females may also be more vulnerable to being trapped by predators in the nest cavity. Here, nest predation was more common at our forest study sites, especially during second broods (mainly due to the edible dormouse *Glis glis* ; our unpublished data), and such events often also result in the death of the female parent. Therefore, while the mechanisms leading to the slight sex‐specific differences in apparent survival may differ between our urban and forest study sites, our findings suggest that the combined effects of these factors ultimately balance out between habitats. The net difference resulted in the slightly higher survival prospects for males, a pattern that has been observed in many previous studies.

### 4.5 Annual Variation in Apparent Survival

According to our models, apparent survival of adults varied widely among years, ranging between 0.25 and 0.66 in the combined models. Our new estimates are consistent with what former studies have reported for other populations of great tits, where researchers estimated annual survival of adult birds to be between 0.20 and 0.60 in Dutch woodland populations (Perdeck et al. 2000) and between 0.12 and 0.68 in a British nationwide study (Robinson et al. 2007). In the site‐specific model sets, a high year‐to‐year variation in apparent survival was present in the supported models in Veszprém (urban), Balatonfüred (urban), and Szentgál (forest), but not in Vilma‐puszta (forest). However, the relatively low annual sample sizes we were able to use for Vilma‐puszta can hinder the detection of a presumably low (lower that the other three site) annual variance.

Adult survival could vary among years due to long‐term population trends (Siriwardena et al. 1998) that might be associated with the variation of the population's density. However, this explanation seems less likely in our case since the models consistently failed to support the effect of breeding density on annual survival. Large year‐to‐year fluctuations in survival are a common phenomenon in small‐bodied species of cavity‐nesting birds that may be more sensitive to environmental effects (Healy et al. 2014). Annual changes in the survival of adult birds are highly influenced by complex environmental factors, like disease (Jiménez‐Peñuela et al. 2019; Lawson et al. 2012), prevailing weather conditions (Dybala et al. 2013; Lv et al. 2023) and fluctuations in natural food supply (Callery et al. 2022; Grøtan et al. 2009). Unfortunately, we do not have additional data for these environmental variables throughout the study period to evaluate whether they were involved in generating the annual fluctuations we detected.

### 4.6 Conclusions

In this study, we found unexpected patterns of variation in apparent survival among urban bird populations: lower survival in one urban location compared to forested areas, and higher in the other urban site. Our results indicate substantial variability in avian survival rates across different urban environments, even among nearby sites. Therefore, our study highlights a common limitation in research design that compares only a single pair of urban and rural study sites. Had our analysis been restricted to a single urban location, we could have inaccurately inferred that urbanisation either enhances or diminishes the survival prospects of great tits relative to forested habitats. Our study thus emphasises the critical need for future research to include multiple urban and rural settings (Ouyang et al. 2018), as various local socioeconomic factors could influence the factors governing the annual survival rates of urban birds (Kinnunen et al. 2022). Last, the observed habitat‐related age effect on survival underscores the possible impact of urbanisation on avian senescence processes—an important implication that requires further research to be explored. In conclusion, our result suggests that different factors influence the apparent survival of adult birds in urban than in natural habitats, and it will be a challenge for upcoming studies to identify the key factors and their effects on survival probabilities.

## Author Contributions

**Boglárka Bukor:** conceptualization (equal), data curation (lead), formal analysis (equal), investigation (equal), methodology (equal), visualization (equal), writing – original draft (equal). **Brett K. Sandercock:** formal analysis (equal), investigation (equal), resources (equal), validation (equal), writing – original draft (equal). **Karl L. Evans:** validation (equal), writing – original draft (equal). **Ivett Pipoly:** investigation (equal), writing – original draft (equal). **Krisztina Sándor:** investigation (equal), writing – original draft (equal). **András Liker:** conceptualization (equal), methodology (equal), project administration (equal), resources (equal), supervision (equal), writing – original draft (equal). **Gábor Seress:** conceptualization (equal), methodology (equal), resources (equal), supervision (equal), writing – original draft (equal).

## Conflicts of Interest

The authors declare no conflicts of interest.

## Supporting information


Appendix S1.

## Data Availability

All necessary data files are included in the main file (as “Appendix” section).

## References

[ece371140-bib-0001] Arnold, T. W. 2010. “Uninformative Parameters and Model Selection Using Akaike's Information Criterion.” Journal of Wildlife Management 74, no. 6: 1175–1178. 10.2193/2009-367.

[ece371140-bib-0002] Aronson, M. F. J. , F. A. La Sorte , C. H. Nilon , et al. 2014. “A Global Analysis of the Impacts of Urbanization on Bird and Plant Diversity Reveals Key Anthropogenic Drivers.” Proceedings of the Royal Society B: Biological Sciences 281, no. 1780: 20133330. 10.1098/rspb.2013.3330. PMC4027400 24523278

[ece371140-bib-0004] Baker, P. J. , A. J. Bentley , R. J. Ansell , and S. Harris . 2005. “Impact of Predation by Domestic Cats *Felis catus* in an Urban Area.” Mammal Review 35, no. 3: 302–312. 10.1111/j.1365-2907.2005.00071.x.

[ece371140-bib-0005] Banks, P. B. 2004. “Population Viability Ananlysis in Urban Wildlife Management: Modelling Management Options for Sydney's Quarantined Bandicoots.” In Royal Zoological Society of New South Wales (Urban Wildlife: More Than Meets the Eye), 70–77. Royal Zoological Society of New South Wales.

[ece371140-bib-0006] Bartos Smith, S. , J. E. McKay , J. K. Richardson , A. A. Shipley , and M. T. Murphy . 2016. “Demography of a Ground Nesting Bird in an Urban System: Are Populations Self‐Sustaining?” Urban Ecosystems 19, no. 2: 577–598. 10.1007/s11252-016-0532-6.

[ece371140-bib-0007] Bastianelli, O. , A. Robert , C. Doutrelant , C. de Franceschi , P. Giovannini , and A. Charmantier . 2021. “Identifying Drivers of Spatio‐Temporal Variation in Survival in Four Blue Tit Populations.” Peer Community Journal 1: e11. 10.24072/pcjournal.17.

[ece371140-bib-0008] Batáry, P. , K. Kurucz , M. Suarez‐Rubio , and D. E. Chamberlain . 2018. “Non‐linearities in Bird Responses Across Urbanization Gradients: A Meta‐Analysis.” Global Change Biology 24, no. 3: 1046–1054. 10.1111/gcb.13964. 29080260

[ece371140-bib-0009] Beninde, J. , M. Veith , and A. Hochkirch . 2015. “Biodiversity in Cities Needs Space: A Meta‐Analysis of Factors Determining Intra‐Urban Biodiversity Variation.” Ecology Letters 18, no. 6: 581–592. 10.1111/ele.12427. 25865805

[ece371140-bib-0010] Bichet, C. , F. Brischoux , C. Ribout , C. Parenteau , A. Meillère , and F. Angelier . 2020. “Physiological and Morphological Correlates of Blood Parasite Infection in Urban and Non‐urban House Sparrow Populations.” PLoS One 15, no. 8: e0237170. 10.1371/journal.pone.0237170. 32813710 PMC7437892

[ece371140-bib-0011] Blair, R. B. 1996. “Land Use and Avian Species Diversity Along an Urban Gradient.” Ecological Applications 6, no. 2: 506–519. 10.2307/2269387.

[ece371140-bib-0012] Bonnington, C. , K. J. Gaston , and K. L. Evans . 2013. “Fearing the Feline: Domestic Cats Reduce Avian Fecundity Through Trait‐Mediated Indirect Effects That Increase Nest Predation by Other Species.” Journal of Applied Ecology 50, no. 1: 15–24. 10.1111/1365-2664.12025.

[ece371140-bib-0013] Borhidi, A. 2003. Magyarország növénytársulásai. Akadémiai Kiadó.

[ece371140-bib-0014] Bourne, A. R. , S. J. Cunningham , L. J. Nupen , A. E. McKechnie , and A. R. Ridley . 2022. “No Sex‐Specific Differences in the Influence of High Air Temperatures During Early Development on Nestling Mass and Fledgling Survival in the Southern Pied Babbler (*Turdoides Bicolor*).” Ibis 164, no. 1: 304–312. 10.1111/ibi.12990.

[ece371140-bib-0015] Bradley, C. A. , and S. Altizer . 2007. “Urbanization and the Ecology of Wildlife Diseases.” Trends in Ecology & Evolution 22, no. 2: 95–102. 10.1016/j.tree.2006.11.001. 17113678 PMC7114918

[ece371140-bib-0016] Branston, C. J. , P. Capilla‐Lasheras , C. J. Pollock , K. Griffiths , S. White , and D. M. Dominoni . 2021. “Urbanisation Weakens Selection on the Timing of Breeding and Clutch Size in Blue Tits but Not in Great Tits.” Behavioral Ecology and Sociobiology 75, no. 11: 155. 10.1007/s00265-021-03096-z.

[ece371140-bib-0017] Bukor, B. 2017. Éven Belüli És Évek Közötti Újrafészkelések Gyakoriságának Vizsgálata Erdei És Városi Széncinegék (Parus major) Populációiban. University of Veterinary Sciences.

[ece371140-bib-0018] Bukor, B. , B. Kósa , A. Liker , and G. Seress . 2024. “Winter Field Survey of Bird Feeders in Two Hungarian Cities.” Ornis Hungarica 32, no. 1: 80–95. 10.2478/orhu-2024-0006.

[ece371140-bib-0019] Caizergues, A. E. , A. Grégoire , R. Choquet , S. Perret , and A. Charmantier . 2022. “Are Behaviour and Stress‐Related Phenotypes in Urban Birds Adaptive?” Journal of Animal Ecology 91, no. 8: 1627–1641. 10.1111/1365-2656.13740. 35575101 PMC9540257

[ece371140-bib-0020] Callery, K. R. , J. A. Smallwood , A. R. Hunt , E. R. Snyder , and J. A. Heath . 2022. “Seasonal Trends in Adult Apparent Survival and Reproductive Trade‐Offs Reveal Potential Constraints to Earlier Nesting in a Migratory Bird.” Oecologia 199, no. 1: 91–102. 10.1007/s00442-022-05169-w. 35451650

[ece371140-bib-0021] Chamberlain, D. E. , A. R. Cannon , M. P. Toms , D. I. Leech , B. J. Hatchwell , and K. J. Gaston . 2009. “Avian Productivity in Urban Landscapes: A Review and Meta‐Analysis.” Ibis 151, no. September 2015: 1–18. 10.1111/j.1474-919X.2008.00899.x.

[ece371140-bib-0022] Chatelain, M. , S. Massemin , S. Zahn , E. Kurek , E. Bulska , and M. Szulkin . 2021. “Urban Metal Pollution Explains Variation in Reproductive Outputs in Great Tits and Blue Tits.” Science of the Total Environment 776: 145966. 10.1016/j.scitotenv.2021.145966.

[ece371140-bib-0023] Climate Central . 2021. Hot zones: urban heat islands.

[ece371140-bib-0024] Clobert, J. , C. M. Perrins , R. H. McCleery , and A. G. Gosler . 1988. “Survival Rate in the Great Tit *Parus Major* in Relation to Sex, Age, and Immigration Status.” Journal of Animal Ecology 57, no. 1: 287–306. 10.2307/4779.

[ece371140-bib-0025] Conradie, S. R. , S. M. Woodborne , S. J. Cunningham , and A. E. McKechnie . 2019. “Chronic, Sublethal Effects of High Temperatures Will Cause Severe Declines in Southern African Arid‐Zone Birds During the 21st Century.” Proceedings of the National Academy of Sciences of the United States of America 116, no. 28: 14065–14070. 10.1073/pnas.1821312116. 31235571 PMC6628835

[ece371140-bib-0026] Cooch, E. G. , and G. C. White . 2022. “Program MARK—A Gentle Introduction—Chapter 5.” In Program MARK—A Gentle Introduction. https://www.nativefishlab.net/library/internalpdf/21294.pdf.

[ece371140-bib-0027] Costa, R. A. , T. Eeva , C. Eira , J. Vaqueiro , and J. V. Vingada . 2013. “Assessing Heavy Metal Pollution Using Great Tits ( *Parus major* ): Feathers and Excrements From Nestlings and Adults.” Environmental Monitoring and Assessment 185, no. 6: 5339–5344. 10.1007/s10661-012-2949-6. 23086543

[ece371140-bib-0028] Costantini, D. , T. J. Greives , M. Hau , and J. Partecke . 2014. “Does Urban Life Change Blood Oxidative Status in Birds?” Journal of Experimental Biology 217, no. 17: 2994–2997. 10.1242/jeb.106450. 24948638

[ece371140-bib-0029] Crawford, B. A. , C. T. Moore , T. M. Norton , and J. C. Maerz . 2018. “Integrated Analysis for Population Estimation, Management Impact Evaluation, and Decision‐Making for a Declining Species.” Biological Conservation 222: 33–43. 10.1016/j.biocon.2018.03.023.

[ece371140-bib-0030] De Merling Chapa, M. , A. Courtiol , M. Engler , et al. 2020. “Phantom of the Forest or Successful Citizen? Analysing How Northern Goshawks (*Accipiter Gentilis*) Cope With the Urban Environment.” Royal Society Open Science 7, no. 12: 201356. 10.1098/rsos.201356. 33489280 PMC7813232

[ece371140-bib-0031] Debbage, N. , and J. M. Shepherd . 2015. “The Urban Heat Island Effect and City Contiguity.” Computers, Environment and Urban Systems 54: 181–194. 10.1016/j.compenvurbsys.2015.08.002.

[ece371140-bib-0032] Deilami, K. , M. Kamruzzaman , and Y. Liu . 2018. “Urban Heat Island Effect : A Systematic Review of Spatio‐Temporal Factors, Data, Methods, and Mitigation Measures.” International Journal of Applied Earth Observation and Geoinformation 67: 30–42.

[ece371140-bib-0033] Del Hoyo, C. , and A. Elliott . 2007. Handbook of the Birds of the World, Picathares to Tits and Chickadees. Vol. 12. Lynx editions.

[ece371140-bib-0034] Dybala, K. E. , J. M. Eadie , T. Gardali , N. E. Seavy , and M. P. Herzog . 2013. “Projecting Demographic Responses to Climate Change: Adult and Juvenile Survival Respond Differently to Direct and Indirect Effects of Weather in a Passerine Population.” Global Change Biology 19, no. 9: 2688–2697. 10.1111/gcb.12228. 23606580

[ece371140-bib-0035] Eden, C. 2021. The Impacts of Urbanisation on Blue Tit (Cyanistes Caeruleus) Population Dynamics. University of Glasgow.

[ece371140-bib-0036] Evans, B. , T. B. Ryder , R. Reitsma , A. H. Hurlbert , and P. P. Marra . 2015. “Characterizing Avian Survival Along a Rural‐To‐Urban Land Use Gradient.” Ecology 96, no. 6: 1631–1640. 10.1890/14-0171.1.

[ece371140-bib-0037] Evans, K. L. 2010. Individual Species and Urbanisation, edited by K. J. Gaston . Cambridge University Press.

[ece371140-bib-0038] Evans, K. L. , K. J. Gaston , S. P. Sharp , A. McGowan , M. Simeoni , and B. J. Hatchwell . 2009. “Effects of Urbanisation on Disease Prevalence and Age Structure in Blackbird *Turdus Merula* Populations.” Oikos 118, no. 5: 774–782. 10.1111/j.1600-0706.2008.17226.x.

[ece371140-bib-0039] Forero, M. G. , J. L. Tella , and D. Or . 2001. “Annual Survival Rates of Adult Redmnecked Nightjars *Caprimulgus ruficollis* .” Valverde 1967: 273–277.

[ece371140-bib-0040] Frątczak, M. , P. Indykiewicz , B. Dulisz , et al. 2021. “Lack of Evidence That Bird Feeders Are a Source of Salmonellosis During Winter in Poland.” Animals 11, no. 6: 1–7. 10.3390/ani11061831. PMC8234643 34205243

[ece371140-bib-0041] Gardner, J. L. , E. Rowley , P. De Rebeira , A. De Rebeira , and L. Brouwer . 2017. “Effects of Extreme Weather on Two Sympatric Australian Passerine Bird Species.” Philosophical Transactions of the Royal Society, B: Biological Sciences 372, no. 1723: 1–11. 10.1098/rstb.2016.0148. PMC5434098 28483863

[ece371140-bib-0042] Grade, A. M. , P. S. Warren , and S. B. Lerman . 2022. “Managing Yards for Mammals: Mammal Species Richness Peaks in the Suburbs.” Landscape and Urban Planning 220: 104337. 10.1016/j.landurbplan.2021.104337.

[ece371140-bib-0043] Grøtan, V. , B. E. Sæther , S. Engen , J. H. Van Balen , A. C. Perdeck , and M. E. Visser . 2009. “Spatial and Temporal Variation in the Relative Contribution of Density Dependence, Climate Variation and Migration to Fluctuations in the Size of Great Tit Populations.” Journal of Animal Ecology 78, no. 2: 447–459. 10.1111/j.1365-2656.2008.01488.x. 19302127

[ece371140-bib-0044] Gullett, P. , K. L. Evans , R. A. Robinson , and B. J. Hatchwell . 2014. “Climate Change and Annual Survival in a Temperate Passerine: Partitioning Seasonal Effects and Predicting Future Patterns.” Oikos 123, no. 4: 389–400. 10.1111/j.1600-0706.2013.00620.x.

[ece371140-bib-0045] Harvey, P. H. , P. J. Greenwood , and C. M. Perrins . 1979. “Breeding Area Fidelity of Great Tits ( *Parus major* ).” Journal of Animal Ecology 48, no. 1: 305. 10.2307/4115.

[ece371140-bib-0046] Healy, K. , T. Guillerme , S. Finlay , et al. 2014. “Ecology and Mode‐Of‐Life Explain Lifespan Variation in Birds and Mammals.” Royal Society 281, no. 1784: 20140298. 10.1098/rspb.2014.0298. PMC4043093 24741018

[ece371140-bib-0047] Horak, P. , and J.‐D. Lebreton . 1998. “Survival of Adult Great Tits Parus Major in Relation to Sex and Habitat; a Comparison of Urban and Rural Populations.” Ibis 140, no. 2: 205–209. 10.1111/j.1474-919X.1998.tb04380.x.

[ece371140-bib-0048] Ibáñez‐Álamo, J. D. , E. Rubio , Y. Benedetti , and F. Morelli . 2016. “Global Loss of Avian Evolutionary Uniqueness in Urban Areas.” Global Change Biology 23, no. 8: 2990–2998. 10.1111/gcb.13567. 27859999

[ece371140-bib-0049] Jarrett, C. , L. L. Powell , H. McDevitt , B. Helm , and A. J. Welch . 2020. “Bitter Fruits of Hard Labour: Diet Metabarcoding and Telemetry Reveal That Urban Songbirds Travel Further for Lower‐Quality Food.” Oecologia 193, no. 2: 377–388. 10.1007/s00442-020-04678-w. 32533359 PMC7320956

[ece371140-bib-0050] Jiménez‐Peñuela, J. , M. Ferraguti , J. Martínez‐de la Puente , R. Soriguer , and J. Figuerola . 2019. “Urbanization and Blood Parasite Infections Affect the Body Condition of Wild Birds.” Science of the Total Environment 651: 3015–3022. 10.1016/j.scitotenv.2018.10.203. 30463151

[ece371140-bib-0051] Juárez, R. , V. Ruiz‐Gutiérrez , and L. Sandoval . 2022. “Surviving in Cities: The Case of a Year‐Round Territorial Bird in the Neotropics.” Journal of Urban Ecology 8, no. 1: 1–7. 10.1093/jue/juac006.

[ece371140-bib-0052] Kark, S. , A. Iwaniuk , A. Schalimtzek , and E. Banker . 2007. “Living in the City: Can Anyone Become an ‘Urban Exploiter’?” Journal of Biogeography 34, no. 4: 638–651. 10.1111/j.1365-2699.2006.01638.x.

[ece371140-bib-0053] Kinnunen, R. P. , K. C. Fraser , C. Schmidt , and C. J. Garroway . 2022. “The Socioeconomic Status of Cities Covaries With Avian Life‐History Strategies.” Ecosphere 13, no. 2: 1–12. 10.1002/ecs2.3918.38988721

[ece371140-bib-0054] Könczey, R. , L. Tóth , and J. Török . 1997. “Site Fidelity of Great and Blue Tit in Pilis‐Visegrád Mountains.” Opuscula Zoologica: 103–111.

[ece371140-bib-0055] Lawson, B. , R. A. Robinson , K. M. Colvile , et al. 2012. “The Emergence and Spread of Finch Trichomonosis in the British Isles.” Philosophical Transactions of the Royal Society, B: Biological Sciences 367, no. 1604: 2852–2863. 10.1098/rstb.2012.0130. PMC3427565 22966140

[ece371140-bib-0056] Loss, S. R. , and P. P. Marra . 2017. “Population Impacts of Free‐Ranging Domestic Cats on Mainland Vertebrates.” Frontiers in Ecology and the Environment 15, no. 9: 502–509. 10.1002/fee.1633.

[ece371140-bib-0057] Loss, S. R. , T. Will , and P. P. Marra . 2015. “Direct Mortality of Birds From Anthropogenic Causes.” Annual Review of Ecology, Evolution, and Systematics 46: 99–120. 10.1146/annurev-ecolsys-112414-054133.

[ece371140-bib-0058] Lv, L. , M. Van De Pol , H. L. Osmond , Y. Liu , A. Cockburn , and L. E. B. Kruuk . 2023. “Winter Mortality of a Passerine Bird Increases Following Hotter Summers and During Winters With Higher Maximum Temperatures.” Ecology 9, no. 1: 1–14. 10.1126/sciadv.abm0197. PMC9812369 36599000

[ece371140-bib-0059] Marciniak, B. , J. Nadolski , M. Nowakowka , B. Loga , and J. Banura . 2007. “Habitat and Annual Variation in Arthropod Abundance Affects Blue Tit *Cyanistes Caeruleus* Reproduction.” Acta Ornithologica 42, no. 1: 53–62. 10.3161/068.042.0113.

[ece371140-bib-0060] Marzluff, J. M. 2017. “A Decadal Review of Urban Ornithology and a Prospectus for the Future.” Ibis 159, no. 1: 1–13. 10.1111/ibi.12430.

[ece371140-bib-0061] Masoero, G. , A. Tamietti , G. Boano , and E. Caprio . 2016. “Apparent Constant Adult Survival of a Sand Martin *Riparia Riparia* Population in Relation to Climatic Variables.” Ardea 104, no. 3: 253–262. 10.5253/arde.v104i3.a1.

[ece371140-bib-0062] McKechnie, A. E. , I. A. Rushworth , F. Myburgh , and S. J. Cunningham . 2021. “Mortality Among Birds and Bats During an Extreme Heat Event in Eastern South Africa.” Austral Ecology 46, no. 4: 687–691. 10.1111/aec.13025.

[ece371140-bib-0063] Millsap, B. A. 2018. “Demography and Metapopulation Dynamics of an Urban Cooper's Hawk Subpopulation.” Condor 120, no. 1: 63–80. 10.1650/CONDOR-17-124.1.

[ece371140-bib-0064] Morozov, N. S. 2022. “The Role of Predators in Shaping Urban Bird Populations: 2. Is Predationp Pressure Increased or Decreased in Urban Landscapes?” Biology Bulletin 49, no. 8: 1081–1104. 10.1134/S106235902208012X.

[ece371140-bib-0065] Murgui, E. , and M. Hedblom . 2017. Ecology and Conservation of Birds in Urban Environments. Springer International Publishing. 10.1007/978-3-319-43314-1.

[ece371140-bib-0066] Nadolski, J. , B. Marciniak , B. Loga , M. Michalski , and J. Bańbura . 2021. “Long‐Term Variation in the Timing and Height of Annual Peak Abundance of Caterpillars in Tree Canopies: Some Effects on a Breeding Songbird.” Ecological Indicators 121: 107120. 10.1016/j.ecolind.2020.107120.

[ece371140-bib-0067] Nagy, G. , and E. Domokos . 2019. Sebezhetőségek és kockázat Veszprém megyében. University of Pannonia. 10.31852/emf.31.2019.087.091.

[ece371140-bib-0068] Orell, M. , and E. J. Belda . 2002. “Delayed Cost of Reproduction and Senescence in the Willow Tit Parus Montanus.” Journal of Animal Ecology 71, no. 1: 55–64. 10.1046/j.0021-8790.2001.00575.x.

[ece371140-bib-0069] Ouyang, J. Q. , C. Isaksson , C. Schmidt , P. Hutton , F. Bonier , and D. Dominoni . 2018. “A New Framework for Urban Ecology: An Integration of Proximate and Ultimate Responses to Anthropogenic Change.” Integrative and Comparative Biology 58, no. 5: 915–928. 10.1093/icb/icy110. 30376106 PMC6204990

[ece371140-bib-0070] Perdeck, A. C. , M. E. Visser , and J. H. Van Balen . 2000. “Great Tit *Parus major* Survival and the Beech Crop Cycle.” Ardea 88: 99–108.

[ece371140-bib-0071] Pharr, L. D. , C. B. Cooper , B. Evans , et al. 2023. “Using Citizen Science Data to Investigate Annual Survival Rates of Resident Birds in Relation to Noise and Light Pollution.” Urban Ecosystems 26: 1629–1637. 10.1007/s11252-023-01403-2.

[ece371140-bib-0072] Phillips, J. N. , K. E. Gentry , E. Derryberry , and D. A. Luther . 2018. “Surviving in the City: Higher Apparent Survivial for Urban Birds but Worse Condition on Noisy Territories.” Ecosphere 9, no. 9: e02440. 10.1002/ecs2.2440.

[ece371140-bib-0073] Pipoly, I. , V. Bókony , M. Kirkpatrick , P. F. Donald , T. Székely , and A. Liker . 2015. “The Genetic Sex‐Determination System Predicts Adult Sex Ratios in Tetrapods.” Nature 527, no. 7576: 91–94. 10.1038/nature15380. 26444239

[ece371140-bib-0074] Pipoly, I. , B. Preiszner , K. Sándor , C. Sinkovics , G. Seress , and E. Vincze . 2020. “Effects of Extreme Hot Weather on the Reproductive Output of Great Tits (*Parus major*) in Urban and Natural Habitats.” BioRxiv 1: 1–18. 10.1101/2020.01.29.924332.

[ece371140-bib-0075] Robb, G. N. , R. A. McDonald , D. E. Chamberlain , and S. Bearhop . 2008. “Food for Thought: Supplementary Feeding as a Driver of Ecological Change in Avian Populations.” Frontiers in Ecology and the Environment 6, no. 9: 476–484. 10.1890/060152.

[ece371140-bib-0076] Robinson, R. A. , S. R. Baillie , and H. Q. P. Crick . 2007. “Weather‐Dependent Survival: Implications of Climate Change for Passerine Population Processes.” Ibis 149, no. 2: 357–364. 10.1111/j.1474-919X.2006.00648.x.

[ece371140-bib-0077] Rodewald, A. D. , and S. D. Gehrt . 2014. Urban Wildlife Conservation: Theory and Practice, 117–147. Springer Science. 10.1007/978-1-4899-7500-3.

[ece371140-bib-0078] Romano, A. , A. Liker , G. Bazzi , R. Ambrosini , A. P. Møller , and D. Rubolini . 2022. “Annual Egg Productivity Predicts Female‐Biased Mortality in Avian Species.” Evolution 76, no. 11: 2553–2565. 10.1111/evo.14623. 36117282 PMC9828124

[ece371140-bib-0079] Salewski, V. , W. M. Hochachka , and W. Fiedler . 2013. “Multiple Weather Factors Affect Apparent Survival of European Passerine Birds.” PLoS One 8, no. 4: 1–10. 10.1371/journal.pone.0059110. PMC3620169 23593131

[ece371140-bib-0080] Saulnier, A. , J. Bleu , G. Lemonnier , P. Uhlrich , S. Zahn , and S. Massemin . 2022. “Does the Urban Environment Act as a Filter on the Individual Quality of Birds?” Birds 3, no. 1: 84–98. 10.3390/birds3010007.

[ece371140-bib-0081] Sepp, T. , K. J. McGraw , A. Kaasik , and M. Giraudeau . 2018. “A Review of Urban Impacts on Avian Life‐History Evolution: Does City Living Lead to Slower Pace of Life?” Global Change Biology 24, no. 4: 1452–1469. 10.1111/gcb.13969. 29168281

[ece371140-bib-0082] Seress, G. , T. Hammer , V. Bókony , et al. 2018. “Impact of Urbanization on Abundance and Phenology of Caterpillars and Consequences for Breeding in an Insectivorous Bird.” Ecological Applications 28, no. 5: 1143–1156. 10.1002/eap.1730. 29679462

[ece371140-bib-0083] Seress, G. , K. Sándor , E. Vincze , et al. 2021. “Contrasting Effects of the COVID‐19 Lockdown on Urban Birds' Reproductive Success in Two Cities.” Scientific Reports 11, no. 1: 1–10. 10.1038/s41598-021-96858-8. 34480051 PMC8417259

[ece371140-bib-0084] Seress, G. , E. Vincze , I. Pipoly , et al. 2017. “Effects of Capture and Video‐Recording on the Behavior and Breeding Success of Great Tits in Urban and Forest Habitats.” Journal of Field Ornithology 88, no. 3: 299–312. 10.1111/jofo.12205.

[ece371140-bib-0085] Sinkovics, C. , G. Seress , I. Pipoly , E. Vincze , and A. Liker . 2021. “Great Tits Feed Their Nestlings With More but Smaller Prey Items and Fewer Caterpillars in Cities Than in Forests.” Scientific Reports 11, no. 1: 24161. 10.1038/s41598-021-03504-4. 34921179 PMC8683465

[ece371140-bib-0086] Sinkovics, C. , G. Seress , I. Pipoly , E. Vincze , and A. Liker . 2023. “Comparison of Nestling Diet Between First and Second Broods of Great Tits *Parus Major* in Urban and Forest Habitats.” Animal Biodiversity and Conservation 46, no. 2: 199–212. 10.32800/abc.2023.46.0199.

[ece371140-bib-0087] Siriwardena, G. M. , S. R. Baillie , and J. D. Wilson . 1998. “Variation in the Survival Rates of Some British Passerines With Respect to Their Population Trends on Farmland.” Bird Study 45, no. 3: 276–292. 10.1080/00063659809461099.

[ece371140-bib-0088] Sloane, S. A. , A. Gordon , and I. D. Connelly . 2022. “Bushtit (*Psaltriparus minimus*) Nestling Mortality Associated With Unprecedented June 2021 Heatwave in Portland, Oregon.” Wilson Journal of Ornithology 134, no. 1: 155–162. 10.1676/21-00080.

[ece371140-bib-0089] Sol, D. , I. Bartomeus , C. González‐Lagos , and S. Pavoine . 2017. “Urbanisation and the Loss of Phylogenetic Diversity in Birds.” Ecology Letters 20, no. 6: 721–729. 10.1111/ele.12769. 28436096

[ece371140-bib-0090] Stracey, C. M. , and S. K. Robinson . 2012. “Are Urban Habitats Ecological Traps for a Native Songbird? Season‐Long Productivity, Apparent Survival, and Site Fi Delity in Urban and Rural Habitats.” Journal of Avian Biology 43: 50–60. 10.1111/j.1600-048X.2011.05520.x.

[ece371140-bib-0091] Streby, H. M. , J. M. Refsnider , and D. E. Andersen . 2014. “Redefining Reproductive Success in Songbirds: Moving Beyond the Nest Success Paradigm.” Auk 131, no. 4: 718–726. 10.1642/auk-14-69.1.

[ece371140-bib-0092] Sumasgutner, P. , A. Koeslag , and A. Amar . 2019. “Senescence in the City: Exploring Ageing Patterns of a Long‐Lived Raptor Across an Urban Gradient.” Journal of Avian Biology 50, no. 12: 1–14. 10.1111/jav.02247.

[ece371140-bib-0093] Svensson, L. , K. Mularney , D. Zetterström , and P. J. Grant . 2009. Collins Bird Guide. The Most Compete Guide of the Birds of Britain and Europe. HarperCollins.

[ece371140-bib-0094] Székely, T. , A. Liker , R. P. Freckleton , C. Fichtel , and P. M. Kappeler . 2014. “Sex‐Biased Survival Predicts Adult Sex Ratio Variation in Wild Birds.” Proceedings of the Royal Society B: Biological Sciences 281, no. 1788: 20140342. 10.1098/rspb.2014.0342. PMC4083783 24966308

[ece371140-bib-0095] Thornton, M. , I. Todd , and S. Roos . 2017. “Breeding Success and Productivity of Urban and Rural Eurasian Sparrowhawks *Accipiter nisus* in Scotland.” Ecoscience 24, no. 3–4: 115–116. 10.1080/11956860.2017.1374322.

[ece371140-bib-0096] Tratalos, J. , R. A. Fuller , K. L. Evans , et al. 2007. “Bird Densities Are Associated With Household Densities.” Global Change Biology 13, no. 8: 1685–1695. 10.1111/j.1365-2486.2007.01390.x.

[ece371140-bib-0097] Tryjanowski, P. , A. P. Møller , F. Morelli , P. Indykiewicz , P. Zduniak , and Ł. Myczko . 2018. “Food Preferences by Birds Using Bird‐Feeders in Winter: A Large‐Scale Experiment.” Avian Research 9, no. 1: 1–6. 10.1186/s40657-018-0111-z.

[ece371140-bib-0098] Tryjanowski, P. , P. Skórka , T. H. Sparks , et al. 2015. “Urban and Rural Habitats Differ in Number and Type of Bird Feeders and in Bird Species Consuming Supplementary Food.” Environmental Science and Pollution Research 22, no. 19: 15097–15103. 10.1007/s11356-015-4723-0. 26003091 PMC4592493

[ece371140-bib-0099] van Heezik, Y. , A. Smyth , A. Adams , and J. Gordon . 2010. “Do Domestic Cats Impose an Unsustainable Harvest on Urban Bird Populations?” Biological Conservation 143, no. 1: 121–130. 10.1016/j.biocon.2009.09.013.

[ece371140-bib-0100] Vaugoyeau, M. , F. Adriaensen , A. Artemyev , et al. 2016. “Interspecific Variation in the Relationship Between Clutch Size, Laying Date and Intensity of Urbanization in Four Species of Hole‐Nesting Birds.” Ecology and Evolution 6, no. 16: 5907–5920. 10.1002/ece3.2335. 27547364 PMC4983601

[ece371140-bib-0101] Verhulst, S. 1998. “Multiple Breeding in the Great Tit, II. The Costs of Rearing a Second Clutch.” Functional Ecology 12, no. 1: 132–140. 10.1046/j.1365-2435.1998.00165.x.

[ece371140-bib-0102] Vincze, E. , V. Bókony , L. Z. Garamszegi , et al. 2021. “Consistency and Plasticity of Risk‐Taking Behaviour Towards Humans at the Nest in Urban and Forest Great Tits, *Parus major* .” Animal Behaviour 179: 161–172. 10.1016/j.anbehav.2021.06.032.

[ece371140-bib-0103] Walthers, A. R. , and C. A. Barber . 2020. “Traffic Noise as a Potential Stressor to Offspring of an Urban Bird, the European Starling.” Journal of Ornithology 161, no. 2: 459–467. 10.1007/s10336-019-01733-z.

[ece371140-bib-0104] Watson, H. , A. A. Cohen , and C. Isaksson . 2015. “A Theoretical Model of the Evolution of Actuarial Senescence Under Environmental Stress.” Experimental Gerontology 71: 80–88. 10.1016/j.exger.2015.08.009. 26335620 PMC4710637

[ece371140-bib-0105] White, G. C. , and K. P. Burnham . 1999. “Program Mark: Survival Estimation From Populations of Marked Animals.” Bird Study 46: S120–S139. 10.1080/00063659909477239.

[ece371140-bib-0106] Whittaker, K. , and J. M. Marzluff . 2013. “Post‐Fledging Mobility in an Urban Landscape.” In Urban Bird Ecology and Conservation, edited by J. M. Marzluff , 182–198. University of California Press. 10.1525/california/9780520273092.003.0012.

[ece371140-bib-0107] Whittaker, K. A. , and J. M. Marzluff . 2009. “Species‐Specific Survival and Relative Habitat Use in an Urban Landscape During the Postfledging Period.” Auk 126, no. 2: 288–299. 10.1525/auk.2009.07136.

[ece371140-bib-0108] Wilby, R. L. , and G. L. W. Perry . 2006. “Climate Change, Biodiversity and the Urban Environment: A Critical Review Based on London, UK.” Progress in Physical Geography 30, no. 1: 73–98. 10.1191/0309133306pp470ra.

[ece371140-bib-0109] Xie, S. , L. M. Romero , Z. W. Htut , and T. J. McWhorter . 2017. “Stress Responses to Heat Exposure in Three Species of Australian Desert Birds.” Physiological and Biochemical Zoology 90, no. 3: 348–358. 10.1086/690484. 28384428

