# Supplementary material for: Site‐Specific Impacts of Urbanisation on Annual Survival of a Forest Bird
Source: Ecol Evol. 2025 May 11;15(5):e71140. doi: 10.1002/ece3.71140 (PMC12066808; doi:10.1002/ece3.71140)
Supplement: Supplementary file 1 — Appendix S1. [file ECE3-15-e71140-s001.docx]

**Appendix**


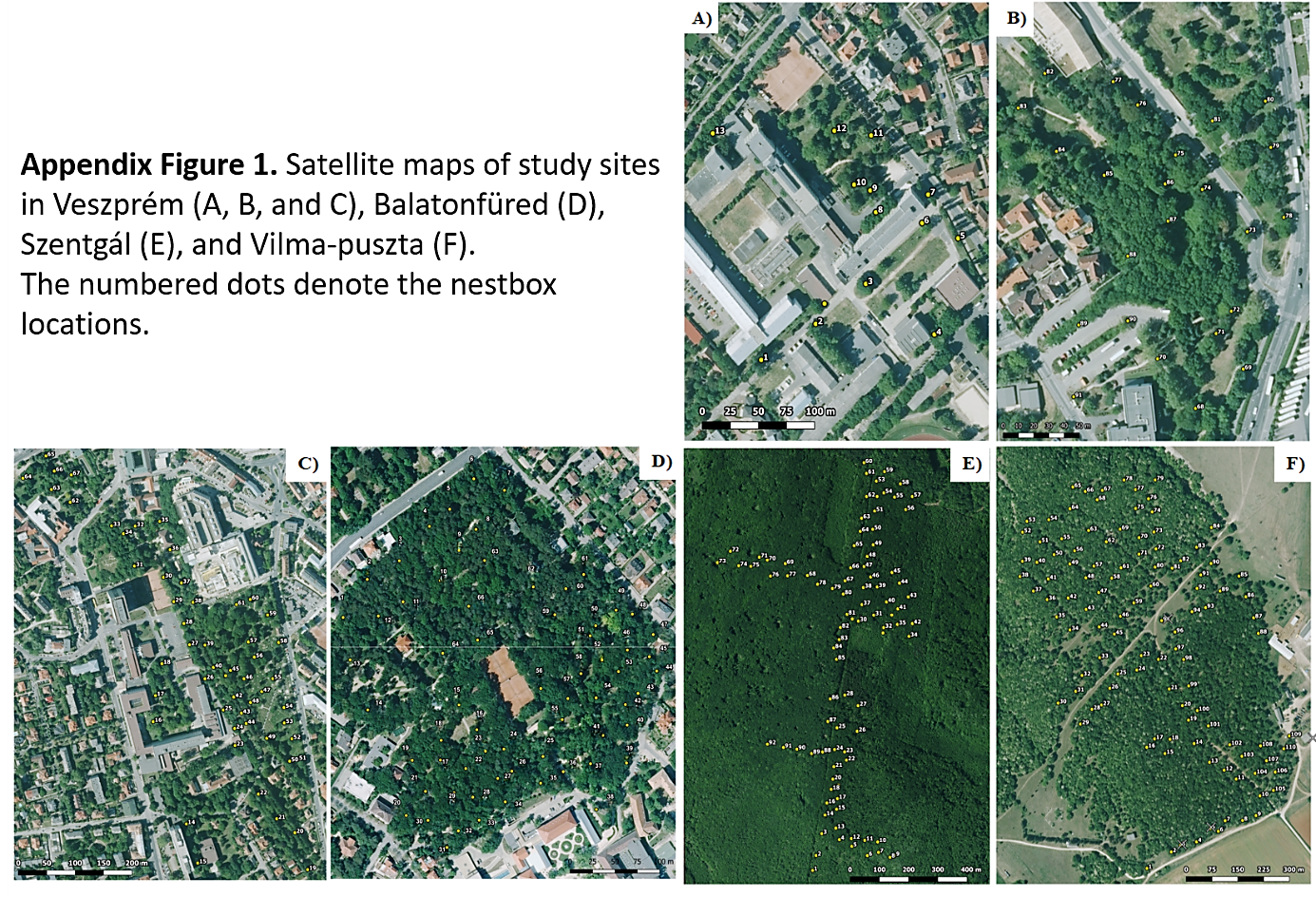


**Appendix Table 1.** Number of available nestboxes in our four study sites, during the study period.

|  | **Veszprém** | **Balatonfüred** | **Szentgál** | **Vilma-puszta** |
| --- | --- | --- | --- | --- |
| **2013** | 81 | 36 | 96 | 89 |
| **2014** | 79 | 65 | 94 | 88 |
| **2015** | 77 | 64 | 94 | 87 |
| **2016** | 77 | 62 | 87 | 86 |
| **2017** | 77 | 68 | 90 | 109 |
| **2018** | 77 | 65 | 88 | 110 |
| **2019** | 77 | 66 | 89 | 110 |
| **2020** | 97 | closed | 85 | 107 |
| **2021** | 97 | closed | 85 | 107 |

**Appendix Table 2.** Total model list from model selection of Cormack–Jolly–Seber capture–recapture analyses for two urban (Veszprém, Balatonfüred) and two forest (Szentgál, Vilma-puszta) breeding populations of great tits between 2013 and 2019. (φ: apparent, annual survival probability; p: re-sighting probability). The supported models (ΔAICc < 2) from the 62 Cormack–Jolly–Seber models possible combinations of parameters are shown in order of AICc. Statistics given for each model include the Akaike information criterion corrected for small sample size (AICc), proportional support of the model (i.e. the AICc weight), number of parameters, likelihood and deviance (deviance: -2logL(θ) i.e. for a given model with parameters θ).

| **Model** | **AICc** | **Delta AICc** | **AICc Weights** | **Model Likelihood** | **Num. Par** | **Deviance** |
| --- | --- | --- | --- | --- | --- | --- |
| **φ(site+time) p(.)}** | **1874.52** | **0** | **0.22107** | **1** | **10** | **294.0457** |
| **φ(site+sex+time) p(.)}** | **1875.116** | **0.596** | **0.1641** | **0.7423** | **11** | **292.6043** |
| **φ(site+time) p(sex)}** | **1876.493** | **1.9732** | **0.08243** | **0.3729** | **11** | **293.9816** |
| φ(site+time+dens) p(.)} | 1876.55 | 2.0305 | 0.0801 | 0.3623 | 11 | 294.0389 |
| φ(site+time) p(dens)} | 1876.555 | 2.0357 | 0.07989 | 0.3614 | 11 | 294.0441 |
| φ(site+sex+time) p(sex)} | 1876.576 | 2.0563 | 0.07907 | 0.3577 | 12 | 292.0239 |
| φ(site+time+sex+dens) p(.)} | 1877.107 | 2.5873 | 0.06063 | 0.2743 | 12 | 292.555 |
| φ(site+time+sex) p(dens)} | 1877.154 | 2.6346 | 0.05922 | 0.2679 | 12 | 292.6023 |
| φ(site+time) p(site)} | 1877.971 | 3.4517 | 0.03936 | 0.178 | 13 | 291.3753 |
| φ(site+time) p(sex+dens)} | 1878.532 | 4.0124 | 0.02973 | 0.1345 | 12 | 293.98 |
| φ(site+sex+time) p(time)} | 1878.534 | 4.0143 | 0.02971 | 0.1344 | 15 | 287.8392 |
| φ(site+sex+time) p(site)} | 1878.662 | 4.1418 | 0.02787 | 0.1261 | 14 | 290.0178 |
| φ(time+dens) p(.)} | 1880.107 | 5.5873 | 0.01353 | 0.0612 | 8 | 303.6972 |
| φ(time+sex+dens) p(.)} | 1880.783 | 6.2633 | 0.00965 | 0.0437 | 9 | 302.3428 |
| φ(site+time) p(time)} | 1880.942 | 6.4221 | 0.00891 | 0.0403 | 15 | 290.247 |
| φ(site+time) p(site+time)} | 1881.735 | 7.2148 | 0.006 | 0.0271 | 17 | 286.9272 |
| φ(site*time)p(.)} | 1883.155 | 8.6357 | 0.00295 | 0.0133 | 25 | 271.7575 |
| φ(time)p(site)} | 1884.596 | 10.0759 | 0.00143 | 0.0065 | 10 | 304.1215 |
| φ(site*time)p(site)} | 1884.675 | 10.1551 | 0.00138 | 0.0062 | 28 | 266.9969 |
| φ(site*time)p(sex)} | 1885.194 | 10.674 | 0.00106 | 0.0048 | 26 | 271.7059 |
| φ(time) p(dens)} | 1886.548 | 12.0284 | 0.00054 | 0.0024 | 8 | 310.1382 |
| φ(time)p(.)} | 1886.778 | 12.2578 | 0.00048 | 0.0022 | 7 | 312.3946 |
| φ(time+sex) p(dens)} | 1887.293 | 12.7733 | 0.00037 | 0.0017 | 9 | 308.8527 |
| φ(time) p(sex+dens)} | 1888.548 | 14.0287 | 0.0002 | 0.0009 | 9 | 310.1082 |
| φ(time)p(sex)} | 1888.773 | 14.2529 | 0.00018 | 0.0008 | 8 | 312.3628 |
| φ(site*time)p(time)} | 1890.126 | 15.6064 | 0.00009 | 0.0004 | 29 | 270.3476 |
| φ(time) p(site+time)} | 1892.86 | 18.3407 | 0.00002 | 0.0001 | 15 | 302.1656 |
| φ(time)p(time)} | 1893.343 | 18.8236 | 0.00002 | 0.0001 | 11 | 310.832 |
| φ(site) p(site+time)} | 1898.262 | 23.7424 | 0 | 0 | 13 | 311.666 |
| φ(site)p(time)} | 1898.31 | 23.7906 | 0 | 0 | 10 | 317.8363 |
| φ(site+sex) p(time)} | 1898.99 | 24.4703 | 0 | 0 | 11 | 316.4787 |
| φ(site+sex) p(site+time)} | 1899.079 | 24.5593 | 0 | 0 | 14 | 310.4353 |
| φ(site*sex) p(site+time)} | 1899.079 | 24.5593 | 0 | 0 | 14 | 310.4353 |
| φ(site+sex+dens) p(.)} | 1900.055 | 25.5357 | 0 | 0 | 6 | 327.6962 |
| φ(site+dens) p(.)} | 1901.478 | 26.958 | 0 | 0 | 6 | 329.1185 |
| φ(site) p(.)} | 1901.605 | 27.0853 | 0 | 0 | 5 | 331.266 |
| φ(site+sex) p(.)} | 1902.215 | 27.6951 | 0 | 0 | 6 | 329.8556 |
| φ(site*sex) p(time)} | 1902.945 | 28.4256 | 0 | 0 | 14 | 314.3016 |
| φ(site) p(dens)} | 1903.597 | 29.0771 | 0 | 0 | 6 | 331.2376 |
| φ(site)p(sex)} | 1903.618 | 29.098 | 0 | 0 | 6 | 331.2584 |
| φ(site)p(site)} | 1903.898 | 29.3784 | 0 | 0 | 8 | 327.4883 |
| φ(site+sex) p(sex)} | 1903.91 | 29.3898 | 0 | 0 | 7 | 329.5267 |
| φ(site+sex) p(dens)} | 1904.214 | 29.6943 | 0 | 0 | 7 | 329.8312 |
| φ(.) p(site+time)} | 1904.497 | 29.9773 | 0 | 0 | 10 | 324.023 |
| φ(site+sex) p(site)} | 1904.608 | 30.0882 | 0 | 0 | 9 | 326.1677 |
| φ(sex) p(site+time)} | 1905.534 | 31.0138 | 0 | 0 | 11 | 323.0222 |
| φ(site) p(sex+dens)} | 1905.613 | 31.0937 | 0 | 0 | 7 | 331.2305 |
| φ(site*sex) p(.)} | 1906.011 | 31.4913 | 0 | 0 | 9 | 327.5707 |
| φ(dens) p(.)} | 1906.36 | 31.8403 | 0 | 0 | 3 | 340.0511 |
| φ(sex+dens) p(.)} | 1907.033 | 32.5129 | 0 | 0 | 4 | 338.7103 |
| φ(site*sex) p(sex)} | 1907.735 | 33.2157 | 0 | 0 | 10 | 327.2613 |
| φ(site*sex) p(site)} | 1908.367 | 33.8471 | 0 | 0 | 12 | 323.8147 |
| φ(.)p(site)} | 1911.372 | 36.852 | 0 | 0 | 5 | 341.0327 |
| φ(.)p(time)} | 1911.479 | 36.9596 | 0 | 0 | 7 | 337.0964 |
| φ(sex)p(site)} | 1912.264 | 37.7446 | 0 | 0 | 6 | 339.9051 |
| φ(sex)p(time)} | 1912.339 | 37.8197 | 0 | 0 | 8 | 335.9296 |
| φ(sex) p(dens)} | 1914.848 | 40.3287 | 0 | 0 | 4 | 346.5262 |
| φ(.)p(.)} | 1915.181 | 40.6613 | 0 | 0 | 2 | 350.8823 |
| φ(sex)p(.)} | 1915.938 | 41.4179 | 0 | 0 | 3 | 349.6287 |
| φ(.)p(sex)} | 1917.191 | 42.6714 | 0 | 0 | 3 | 350.8823 |
| φ(sex)p(sex)} | 1917.739 | 43.2196 | 0 | 0 | 4 | 349.4171 |
| φ(site*time*sex). p(site*time*sex)} | 1920.375 | 45.855 | 0 | 0 | 70 | 211.2661 |

**Appendix Table 3.** Total model list from model selection of Cormack–Jolly–Seber capture–recapture analyses for data on adult great in Veszprém (city) breeding populations between 2013 and 2021. (φ: apparent, annual survival probability; p: re-sighting probability). The supported models (ΔAICc < 2) from the 57 Cormack–Jolly–Seber models possible combinations of parameters are shown in order of AICc. Statistics given for each model include the Akaike information criterion corrected for small sample size (AICc), proportional support of the model (i.e. the AICc weight), number of parameters, likelihood and deviance (deviance: -2logL(θ) i.e. for a given model with parameters θ). The apparent annual survival of 2Y individuals in their first year (φ1) was considered to differ from that of 2+ individuals, but we did not make this distinction in subsequent years, i.e. when 2Y birds moved to the 2+ age class (φ2).

| Model | AICc | Delta AICc | AICc Weights | Model Likelihood | Num. Par | Deviance |
| --- | --- | --- | --- | --- | --- | --- |
| φ1(t), φ2(.), p(.) | 935.0298 | 0 | 0.27896 | 1 | 10 | 173.3684 |
| φ1(sex+t), φ2(.), p(.) | 935.7681 | 0.7383 | 0.19285 | 0.6913 | 11 | 172.0299 |
| φ1(t), φ2(.), p(sex) | 936.5962 | 1.5664 | 0.12747 | 0.4569 | 11 | 172.8581 |
| φ1(t), φ2(sex), p(.) | 937.0992 | 2.0694 | 0.09912 | 0.3553 | 11 | 173.3611 |
| φ1(sex+t), φ2(.), p(sex) | 937.6685 | 2.6387 | 0.07457 | 0.2673 | 12 | 171.8464 |
| φ1(sex+t), φ2(sex), p(.) | 937.8339 | 2.8041 | 0.06865 | 0.2461 | 12 | 172.0118 |
| φ1(t), φ2(sex), p(sex) | 938.6495 | 3.6197 | 0.04566 | 0.1637 | 12 | 172.8274 |
| φ1(sex+t), φ2(sex), p(sex) | 939.7247 | 4.6949 | 0.02667 | 0.0956 | 13 | 171.8114 |
| φ(t). p(.) | 941.2639 | 6.2341 | 0.01235 | 0.0443 | 9 | 181.6722 |
| φ1(.), φ2(.), p(.) | 941.487 | 6.4572 | 0.01105 | 0.0396 | 3 | 194.1647 |
| φ(.). p(.) | 941.7867 | 6.7569 | 0.00951 | 0.0341 | 2 | 196.4849 |
| φ1(sex), φ2(.), p(.) | 942.9061 | 7.8763 | 0.00544 | 0.0195 | 4 | 193.5563 |
| φ1(.), φ2(.), p(sex) | 943.0838 | 8.054 | 0.00497 | 0.0178 | 4 | 193.734 |
| φ(t). p(sex) | 943.0966 | 8.0668 | 0.00494 | 0.0177 | 10 | 181.4352 |
| φ(t+sex). p(.) | 943.2942 | 8.2644 | 0.00448 | 0.0161 | 10 | 181.6329 |
| φ(.). p(sex) | 943.4945 | 8.4647 | 0.00405 | 0.0145 | 3 | 196.1722 |
| φ1(.), φ2(sex), p(.) | 943.5013 | 8.4715 | 0.00404 | 0.0145 | 4 | 194.1516 |
| φ1(.), φ2(.), p(t) | 943.6523 | 8.6225 | 0.00374 | 0.0134 | 10 | 181.991 |
| φ(sex). p(.) | 943.778 | 8.7482 | 0.00351 | 0.0126 | 3 | 196.4557 |
| φ1(sex), φ2(.), p(sex) | 944.7452 | 9.7154 | 0.00217 | 0.0078 | 5 | 193.3611 |
| φ1(sex), φ2(.), p(t) | 945.0589 | 10.0291 | 0.00185 | 0.0066 | 11 | 181.3208 |
| φ1(.), φ2(sex), p(sex) | 945.0743 | 10.0445 | 0.00184 | 0.0066 | 5 | 193.6902 |
| φ(t+sex). p(sex) | 945.1644 | 10.1346 | 0.00176 | 0.0063 | 11 | 181.4262 |
| φ(.). p(t) | 945.4168 | 10.387 | 0.00155 | 0.0056 | 9 | 185.8251 |
| φ1(t), φ2(.), p(t) | 945.4406 | 10.4108 | 0.00153 | 0.0055 | 17 | 169.0893 |
| φ(sex). p(sex) | 945.5211 | 10.4913 | 0.00147 | 0.0053 | 4 | 196.1714 |
| φ1(.), φ2(sex), p(t) | 945.7209 | 10.6911 | 0.00133 | 0.0048 | 11 | 181.9828 |
| φ(.). p(t+sex) | 946.4213 | 11.3915 | 0.00094 | 0.0034 | 10 | 184.7599 |
| φ(sex). p(t) | 947.4631 | 12.4333 | 0.00056 | 0.002 | 10 | 185.8017 |
| φ1(t), φ2(sex), p(t) | 947.5592 | 12.5294 | 0.00053 | 0.0019 | 18 | 169.0799 |
| φ1(sex*t), φ2(.), p(.) | 948.3093 | 13.2795 | 0.00036 | 0.0013 | 18 | 169.83 |
| φ(sex). p(t+sex) | 948.4658 | 13.436 | 0.00034 | 0.0012 | 11 | 184.7277 |
| φ1(.), φ2(t), p(.) | 949.4431 | 14.4133 | 0.00021 | 0.0008 | 10 | 187.7818 |
| φ1(.), φ2(t), p(t) | 949.819 | 14.7892 | 0.00017 | 0.0006 | 17 | 173.4677 |
| φ1(sex*t), φ2(.), p(sex) | 950.2913 | 15.2615 | 0.00014 | 0.0005 | 19 | 169.6766 |
| φ1(sex*t), φ2(sex), p(.) | 950.4271 | 15.3973 | 0.00013 | 0.0005 | 19 | 169.8124 |
| φ(t). p(t) | 950.8523 | 15.8225 | 0.0001 | 0.0004 | 15 | 178.7347 |
| φ1(sex), φ2(t), p(.) | 950.8981 | 15.8683 | 0.0001 | 0.0004 | 11 | 187.16 |
| φ1(.), φ2(t), p(sex) | 951.1898 | 16.16 | 0.00009 | 0.0003 | 11 | 187.4517 |
| φ1(sex), φ2(t), p(t) | 951.262 | 16.2322 | 0.00008 | 0.0003 | 18 | 172.7827 |
| φ1(.), φ2(sex+t), p(.) | 951.5177 | 16.4879 | 0.00007 | 0.0003 | 11 | 187.7796 |
| φ1(.), φ2(sex+t), p(t) | 951.9458 | 16.916 | 0.00006 | 0.0002 | 18 | 173.4666 |
| φ1(sex*t), φ2(sex), p(sex) | 952.4018 | 17.372 | 0.00005 | 0.0002 | 20 | 169.6441 |
| φ1(sex), φ2(t), p(sex) | 952.8562 | 17.8264 | 0.00004 | 0.0001 | 12 | 187.0341 |
| φ(t+sex). p(t) | 952.9256 | 17.8958 | 0.00004 | 0.0001 | 16 | 178.6948 |
| φ1(.), φ2(sex+t), p(sex) | 953.258 | 18.2282 | 0.00003 | 0.0001 | 12 | 187.4359 |
| φ(t*sex). p(.) | 953.6241 | 18.5943 | 0.00003 | 0.0001 | 17 | 177.2728 |
| φ(t). p(t+sex) | 954.2158 | 19.186 | 0.00002 | 0.0001 | 17 | 177.8645 |
| φ(t*sex). p(sex) | 955.4824 | 20.4526 | 0.00001 | 0 | 18 | 177.0031 |
| φ(t+sex). p(t+sex) | 956.3141 | 21.2843 | 0.00001 | 0 | 18 | 177.8349 |
| φ1(.), φ2(sex*t), p(t) | 958.7358 | 23.706 | 0 | 0 | 24 | 167.3306 |
| φ1(sex*t), φ2(.), p(t) | 959.1372 | 24.1074 | 0 | 0 | 25 | 165.5509 |
| φ1(.), φ2(sex*t), p(.) | 960.018 | 24.9882 | 0 | 0 | 18 | 181.5387 |
| φ1(sex*t), φ2(sex), p(t) | 961.3055 | 26.2757 | 0 | 0 | 26 | 165.5303 |
| φ1(.), φ2(sex*t), p(sex) | 961.5853 | 26.5555 | 0 | 0 | 19 | 180.9706 |
| φ(t*sex). p(t) | 963.5728 | 28.543 | 0 | 0 | 23 | 174.3409 |
| φ1(sex), φ2(sex*t), p(sex*t) | 970.6115 | 35.5817 | 0 | 0 | 34 | 157.0418 |

**Appendix Table 4.** Total model list from model selection of Cormack–Jolly–Seber capture–recapture analyses for data on adult great in Balatonfüred (city) breeding populations between 2013 and 2019. (φ: apparent, annual survival probability; p: re-sighting probability). The supported models (ΔAICc < 2) from the 57 Cormack–Jolly–Seber models possible combinations of parameters are shown in order of AICc. Statistics given for each model include the Akaike information criterion corrected for small sample size (AICc), proportional support of the model (i.e. the AICc weight), number of parameters, likelihood and deviance (deviance: -2logL(θ) i.e. for a given model with parameters θ). The apparent annual survival of 2Y individuals in their first year (φ1) was considered to differ from that of 2+ individuals, but we did not make this distinction in subsequent years, i.e. when 2Y birds moved to the 2+ age class (φ2).

| Model | AICc | Delta AICc | AICc Weights | Model Likelihood | Num. Par | Deviance |
| --- | --- | --- | --- | --- | --- | --- |
| φ1(.), φ2(sex*t), p(.) | 242.2704 | 0 | 0.46611 | 1 | 10 | 51.4773 |
| φ1(sex), φ2(.), p(t) | 244.047 | 1.7766 | 0.19174 | 0.4114 | 6 | 62.0954 |
| φ1, φ2(sex*t), p(t) | 245.3373 | 3.0669 | 0.10058 | 0.2158 | 13 | 47.6248 |
| φ1(.), φ2(sex*t), p(sex) | 246.8278 | 4.5574 | 0.04774 | 0.1024 | 12 | 51.4503 |
| φ1(.), φ2(.), p(t) | 247.5989 | 5.3285 | 0.03247 | 0.0697 | 6 | 65.6473 |
| φ(.). p(t) | 247.9167 | 5.6463 | 0.0277 | 0.0594 | 4 | 70.2298 |
| φ1(sex), φ2(.), p(.) | 247.9998 | 5.7294 | 0.02657 | 0.057 | 4 | 70.3129 |
| φ1(sex), φ2(.), p(sex) | 249.5506 | 7.2802 | 0.01224 | 0.0263 | 5 | 69.7439 |
| φ1(.), φ2(sex), p(t) | 249.7191 | 7.4487 | 0.01125 | 0.0241 | 7 | 65.5968 |
| φ1(.), φ2(.), p(.) | 249.8919 | 7.6215 | 0.01032 | 0.0221 | 3 | 74.3 |
| φ(.). p(t+sex) | 249.9208 | 7.6504 | 0.01017 | 0.0218 | 5 | 70.1141 |
| φ(sex). p(t+sex) | 251.4228 | 9.1524 | 0.0048 | 0.0103 | 6 | 69.4712 |
| φ1(.), φ2(.), p(sex) | 251.5019 | 9.2315 | 0.00461 | 0.0099 | 4 | 73.815 |
| φ1(.), φ2(sex), p(.) | 251.8012 | 9.5308 | 0.00397 | 0.0085 | 4 | 74.1143 |
| φ(.). p(.) | 252.1707 | 9.9003 | 0.0033 | 0.0071 | 2 | 78.6493 |
| φ1(sex), φ2(t), p(.) | 253.1006 | 10.8302 | 0.00207 | 0.0044 | 9 | 64.5581 |
| φ1(sex*t), φ2(.), p(t) | 253.1377 | 10.8673 | 0.00204 | 0.0044 | 14 | 53.0611 |
| φ1(.), φ2(sex), p(sex) | 253.3762 | 11.1058 | 0.00181 | 0.0039 | 5 | 73.5695 |
| φ(t). p(.) | 253.5842 | 11.3138 | 0.00163 | 0.0035 | 7 | 69.4619 |
| φ(sex). p(.) | 253.8503 | 11.5799 | 0.00143 | 0.0031 | 3 | 78.2584 |
| φ(.). p(sex) | 254.2412 | 11.9708 | 0.00117 | 0.0025 | 3 | 78.6492 |
| φ1(sex+t), φ2(.), p(.) | 254.2543 | 11.9839 | 0.00116 | 0.0025 | 9 | 65.7118 |
| φ(t). p(t) | 254.5489 | 12.2785 | 0.00101 | 0.0022 | 9 | 66.0064 |
| φ1(sex+t), φ2(.), p(sex) | 254.8024 | 12.532 | 0.00089 | 0.0019 | 10 | 64.0093 |
| φ1(sex), φ2(t), p(sex) | 254.8498 | 12.5794 | 0.00086 | 0.0018 | 10 | 64.0567 |
| φ1(.), φ2(t), p(.) | 254.85 | 12.5796 | 0.00086 | 0.0018 | 8 | 68.5309 |
| φ1(.), φ2(t), p(t) | 254.9606 | 12.6902 | 0.00082 | 0.0018 | 11 | 61.8894 |
| φ(t+sex). p(.) | 255.2631 | 12.9927 | 0.0007 | 0.0015 | 8 | 68.944 |
| φ1(sex*t), φ2(sex), p(t) | 255.2817 | 13.0113 | 0.0007 | 0.0015 | 15 | 52.8112 |
| φ1(t), φ2(.), p(t) | 255.325 | 13.0546 | 0.00068 | 0.0015 | 10 | 64.532 |
| φ(t). p(sex) | 255.7689 | 13.4985 | 0.00055 | 0.0012 | 8 | 69.4498 |
| φ(sex). p(sex) | 255.8574 | 13.587 | 0.00052 | 0.0011 | 4 | 78.1704 |
| φ1(sex), φ2(t), p(t) | 256.047 | 13.7766 | 0.00048 | 0.001 | 13 | 58.3345 |
| φ(sex), p(t) | 256.0508 | 13.7804 | 0.00047 | 0.001 | 8 | 69.7318 |
| φ1(sex+t), φ2(sex), p(.) | 256.1359 | 13.8655 | 0.00045 | 0.001 | 10 | 65.3428 |
| φ(t+sex). p(t) | 256.1541 | 13.8837 | 0.00045 | 0.001 | 10 | 65.361 |
| φ1(t), φ2(.), p(.) | 256.1636 | 13.8932 | 0.00045 | 0.001 | 8 | 69.8445 |
| φ1(.), φ2(t), p(sex) | 256.5615 | 14.2911 | 0.00037 | 0.0008 | 9 | 68.0189 |
| φ(t). p(t+sex) | 256.7014 | 14.431 | 0.00034 | 0.0007 | 10 | 65.9083 |
| φ1(.), φ2(sex+t), p(.) | 256.8423 | 14.5719 | 0.00032 | 0.0007 | 9 | 68.2998 |
| φ1(sex+t), φ2(sex), p(sex) | 256.9419 | 14.6715 | 0.0003 | 0.0006 | 11 | 63.8706 |
| φ(t+sex). p(t+sex) | 257.098 | 14.8276 | 0.00028 | 0.0006 | 11 | 64.0268 |
| φ(t+sex). p(sex) | 257.4136 | 15.1432 | 0.00024 | 0.0005 | 9 | 68.8711 |
| φ1(t), φ2(sex), p(t) | 257.5255 | 15.2551 | 0.00023 | 0.0005 | 11 | 64.4542 |
| φ1(t), φ2(.), p(sex) | 258.0936 | 15.8232 | 0.00017 | 0.0004 | 9 | 69.5511 |
| φ1(t), φ2(sex), p(.) | 258.1769 | 15.9065 | 0.00016 | 0.0003 | 9 | 69.6344 |
| φ1(.), φ2(sex+t), p(sex) | 258.4383 | 16.1679 | 0.00014 | 0.0003 | 10 | 67.6452 |
| φ1(sex*t), φ2(sex), p(.) | 258.4711 | 16.2007 | 0.00014 | 0.0003 | 14 | 58.3945 |
| φ1(sex*t), φ2(.), p(sex) | 258.7138 | 16.4434 | 0.00013 | 0.0003 | 14 | 58.6372 |
| φ1(sex*t), φ2(.), p(.) | 258.8828 | 16.6124 | 0.00012 | 0.0003 | 14 | 58.8062 |
| φ1(.), φ2(sex+t), p(t) | 259.565 | 17.2946 | 0.00008 | 0.0002 | 13 | 61.8525 |
| φ1(t), φ2(sex), p(sex) | 260.0652 | 17.7948 | 0.00006 | 0.0001 | 10 | 69.2721 |
| φ(t*sex). p(.) | 260.3197 | 18.0493 | 0.00006 | 0.0001 | 13 | 62.6072 |
| φ1(sex*t), φ2(sex), p(sex) | 260.772 | 18.5016 | 0.00004 | 0.0001 | 15 | 58.3015 |
| φ(t*sex). p(t) | 261.3769 | 19.1065 | 0.00003 | 0.0001 | 15 | 58.9065 |
| φ(t*sex). p(sex) | 262.502 | 20.2316 | 0.00002 | 0 | 14 | 62.4254 |
| φ1(sex), φ2(sex*t), p(sex-t) | 264.7937 | 22.5233 | 0.00001 | 0 | 26 | 33.8556 |

**Appendix Table 5.** Total model list from model selection of Cormack–Jolly–Seber capture–recapture analyses for data on adult great in Szentgál (forest) breeding populations between 2013 and 2021. (φ: apparent, annual survival probability; p: re-sighting probability). The supported models (ΔAICc < 2) from the 57 Cormack–Jolly–Seber models possible combinations of parameters are shown in order of AICc. Statistics given for each model include the Akaike information criterion corrected for small sample size (AICc), proportional support of the model (i.e. the AICc weight), number of parameters, likelihood and deviance (deviance: -2logL(θ) i.e. for a given model with parameters θ). The apparent annual survival of 2Y individuals in their first year (φ1) was considered to differ from that of 2+ individuals, but we did not make this distinction in subsequent years, i.e. when 2Y birds moved to the 2+ age class (φ2).

| Model | AICc | Delta AICc | AICc Weights | Model Likelihood | Num. Par | Deviance |
| --- | --- | --- | --- | --- | --- | --- |
| φ(t+sex). p(t) | 754.0277 | 0 | 0.24959 | 1 | 15 | 144.0346 |
| φ(t). p(t) | 754.351 | 0.3233 | 0.21233 | 0.8507 | 14 | 146.4861 |
| φ(t+sex). p(t+sex) | 754.6822 | 0.6545 | 0.17993 | 0.7209 | 17 | 140.4057 |
| φ(t). p(t+sex) | 756.2314 | 2.2037 | 0.08293 | 0.3323 | 16 | 144.1012 |
| φ(t+sex). p(sex) | 756.7505 | 2.7228 | 0.06397 | 0.2563 | 11 | 155.2168 |
| φ(t). p(.) | 756.9323 | 2.9046 | 0.05841 | 0.234 | 9 | 159.5755 |
| φ(t). p(sex) | 757.5241 | 3.4964 | 0.04345 | 0.1741 | 10 | 158.0832 |
| φ(t+sex). p(.) | 757.7448 | 3.7171 | 0.03891 | 0.1559 | 10 | 158.3039 |
| φ(t*sex). p(t) | 761.6126 | 7.5849 | 0.00563 | 0.0226 | 21 | 138.6599 |
| φ1(.), φ2(t), p(.) | 765.3121 | 11.2844 | 0.00088 | 0.0035 | 10 | 165.8712 |
| φ1(.), φ2(sex+t), p(sex) | 765.7314 | 11.7037 | 0.00072 | 0.0029 | 12 | 162.0961 |
| φ1(.), φ2(sex+t), p(.) | 766.0094 | 11.9817 | 0.00062 | 0.0025 | 11 | 164.4756 |
| φ1(.), φ2(t), p(sex) | 766.0696 | 12.0419 | 0.00061 | 0.0024 | 11 | 164.5359 |
| φ1(.), φ2(sex+t), p(t) | 766.0735 | 12.0458 | 0.0006 | 0.0024 | 16 | 153.9433 |
| φ(t*sex). p(.) | 766.0873 | 12.0596 | 0.0006 | 0.0024 | 17 | 151.8109 |
| φ(t*sex). p(sex) | 766.3381 | 12.3104 | 0.00053 | 0.0021 | 18 | 149.9063 |
| φ1(sex), φ2(t), p(.) | 767.2752 | 13.2475 | 0.00033 | 0.0013 | 11 | 165.7415 |
| φ(.). p(t) | 767.7283 | 13.7006 | 0.00026 | 0.001 | 7 | 174.5138 |
| φ1(sex), φ2(t), p(sex) | 767.8119 | 13.7842 | 0.00025 | 0.001 | 12 | 164.1766 |
| φ1(.), φ2(t), p(t) | 767.9131 | 13.8854 | 0.00024 | 0.001 | 16 | 155.7829 |
| φ(sex). p(t) | 768.943 | 14.9153 | 0.00014 | 0.0006 | 8 | 173.6616 |
| φ(.). p(t+sex) | 769.4262 | 15.3985 | 0.00011 | 0.0004 | 8 | 174.1448 |
| φ1(.), φ2(.), p(t) | 769.7951 | 15.7674 | 0.00009 | 0.0004 | 8 | 174.5138 |
| φ1(sex), φ2(t), p(t) | 769.8344 | 15.8067 | 0.00009 | 0.0004 | 17 | 155.5579 |
| φ1(t), φ2(.), p(t) | 770.1573 | 16.1296 | 0.00008 | 0.0003 | 14 | 162.2923 |
| φ(sex). p(t+sex) | 770.3114 | 16.2837 | 0.00007 | 0.0003 | 9 | 172.9546 |
| φ1(.), φ2(sex), p(t) | 771.1061 | 17.0784 | 0.00005 | 0.0002 | 9 | 173.7493 |
| φ1(t), φ2(sex), p(t) | 771.5954 | 17.5677 | 0.00004 | 0.0002 | 15 | 161.6023 |
| φ1(sex), φ2(.), p(t) | 771.7366 | 17.7089 | 0.00004 | 0.0002 | 9 | 174.3798 |
| φ1(.), φ2(sex*t), p(.) | 773.7611 | 19.7334 | 0.00001 | 0 | 18 | 157.3293 |
| φ1(.), φ2(sex*t), p(sex) | 774.1839 | 20.1562 | 0.00001 | 0 | 19 | 155.5877 |
| φ1(sex*t), φ2(.), p(t) | 774.3269 | 20.2992 | 0.00001 | 0 | 19 | 155.7307 |
| φ1(sex*t), φ2(sex), p(t) | 775.7912 | 21.7635 | 0 | 0 | 20 | 155.0214 |
| φ(.). p(.) | 776.3707 | 22.343 | 0 | 0 | 2 | 193.3635 |
| φ1(.), φ2(sex*t), p(t) | 776.5396 | 22.5119 | 0 | 0 | 24 | 146.9819 |
| φ(sex). p(sex) | 776.8603 | 22.8326 | 0 | 0 | 4 | 189.7954 |
| φ(.). p(sex) | 776.9295 | 22.9018 | 0 | 0 | 3 | 191.8976 |
| φ(sex). p(.) | 777.4008 | 23.3731 | 0 | 0 | 3 | 192.3689 |
| φ1(t), φ2(.), p(.) | 778.1685 | 24.1408 | 0 | 0 | 9 | 180.8117 |
| φ1(t), φ2(.), p(sex) | 778.328 | 24.3003 | 0 | 0 | 10 | 178.887 |
| φ1(.), φ2(.), p(.) | 778.3441 | 24.3164 | 0 | 0 | 3 | 193.3123 |
| φ1(t), φ2(sex), p(sex) | 778.5701 | 24.5424 | 0 | 0 | 11 | 177.0364 |
| φ1(.), φ2(.), p(sex) | 778.9361 | 24.9084 | 0 | 0 | 4 | 191.8712 |
| φ1(.), φ2(sex), p(sex) | 779.1482 | 25.1205 | 0 | 0 | 5 | 190.0419 |
| φ1(t), φ2(sex), p(.) | 779.2999 | 25.2722 | 0 | 0 | 10 | 179.859 |
| φ1(.), φ2(sex), p(.) | 779.3561 | 25.3284 | 0 | 0 | 4 | 192.2912 |
| φ1(sex+t), φ2(.), p(.) | 780.0613 | 26.0336 | 0 | 0 | 10 | 180.6203 |
| φ1(sex), φ2(.), p(.) | 780.2705 | 26.2428 | 0 | 0 | 4 | 193.2056 |
| φ1(sex+t), φ2(.), p(sex) | 780.3936 | 26.3659 | 0 | 0 | 11 | 178.8599 |
| φ1(sex), φ2(.), p(sex) | 780.638 | 26.6103 | 0 | 0 | 5 | 191.5317 |
| φ1(sex+t), φ2(sex), p(sex) | 780.6514 | 26.6237 | 0 | 0 | 12 | 177.0161 |
| φ1(sex+t), φ2(sex), p(.) | 781.1736 | 27.1459 | 0 | 0 | 11 | 179.6398 |
| φ1(sex*t), φ2(.), p(.) | 782.115 | 28.0873 | 0 | 0 | 14 | 174.2501 |
| φ1(sex*t), φ2(.), p(sex) | 782.711 | 28.6833 | 0 | 0 | 15 | 172.7179 |
| φ1(sex*t), φ2(sex), p(sex) | 783.0703 | 29.0426 | 0 | 0 | 16 | 170.9401 |
| φ1(sex*t), φ2(sex), p(.) | 783.2705 | 29.2428 | 0 | 0 | 15 | 173.2774 |
| φ1(t*sex), φ2(t*sex), p(t*sex) | 792.7898 | 38.7621 | 0 | 0 | 46 | 112.0685 |

**Appendix Table 6.** Total model list from model selection of Cormack–Jolly–Seber capture–recapture analyses for data on adult great in Vilma-puszta (forest) breeding populations between 2013 and 2021. (φ: apparent, annual survival probability; p: re-sighting probability). The supported models (ΔAICc < 2) from the 57 Cormack–Jolly–Seber models possible combinations of parameters are shown in order of AICc. Statistics given for each model include the Akaike information criterion corrected for small sample size (AICc), proportional support of the model (i.e. the AICc weight), number of parameters, likelihood and deviance (deviance: -2logL(θ) i.e. for a given model with parameters θ). The apparent annual survival of 2Y individuals in their first year (φ1) was considered to differ from that of 2+ individuals, but we did not make this distinction in subsequent years, i.e. when 2Y birds moved to the 2+ age class (φ2).

| Model | AICc | Delta AICc | AICc Weights | Model Likelihood | Num. Par | Deviance |
| --- | --- | --- | --- | --- | --- | --- |
| φ(.). p(t+sex) | 391.3523 | 0 | 0.25012 | 1 | 7 | 126.0293 |
| φ(.). p(t) | 391.7805 | 0.4282 | 0.20191 | 0.8073 | 6 | 128.5718 |
| φ(sex). p(t+sex) | 393.2937 | 1.9414 | 0.09475 | 0.3788 | 8 | 125.8393 |
| φ1(.), φ2(.), p(t) | 393.4976 | 2.1453 | 0.08557 | 0.3421 | 7 | 128.1746 |
| φ1(sex), φ2(.), p(t) | 394.7974 | 3.4451 | 0.04467 | 0.1786 | 8 | 127.343 |
| φ1(.), φ2(sex), p(t) | 395.1698 | 3.8175 | 0.03708 | 0.1483 | 8 | 127.7154 |
| φ(sex). p(t) | 395.1783 | 3.826 | 0.03693 | 0.1477 | 8 | 127.7239 |
| φ1(t), φ2(.), p(t) | 395.2192 | 3.8669 | 0.03618 | 0.1447 | 14 | 114.605 |
| φ(t). p(.) | 395.3868 | 4.0345 | 0.03327 | 0.133 | 9 | 125.7837 |
| φ(t+sex). p(.) | 396.413 | 5.0607 | 0.01992 | 0.0796 | 10 | 124.6436 |
| φ1(t), φ2(.), p(.) | 396.4729 | 5.1206 | 0.01933 | 0.0773 | 10 | 124.7035 |
| φ1(t), φ2(sex), p(t) | 397.1502 | 5.7979 | 0.01378 | 0.0551 | 15 | 114.2787 |
| φ1(sex+t), φ2(.), p(.) | 397.2124 | 5.8601 | 0.01335 | 0.0534 | 11 | 123.259 |
| φ(t). p(sex) | 397.2242 | 5.8719 | 0.01328 | 0.0531 | 10 | 125.4549 |
| φ1(sex*t), φ2(.), p(t) | 397.676 | 6.3237 | 0.01059 | 0.0423 | 20 | 103.2301 |
| φ1(t), φ2(.), p(sex) | 398.006 | 6.6537 | 0.00898 | 0.0359 | 11 | 124.0527 |
| φ1(t), φ2(sex), p(.) | 398.3305 | 6.9782 | 0.00764 | 0.0305 | 11 | 124.3772 |
| φ(t). p(t) | 398.5184 | 7.1661 | 0.00695 | 0.0278 | 13 | 120.1428 |
| φ(t+sex). p(sex) | 398.5751 | 7.2228 | 0.00676 | 0.027 | 11 | 124.6217 |
| φ1(sex*t), φ2(.), p(.) | 398.7438 | 7.3915 | 0.00621 | 0.0248 | 16 | 113.5962 |
| φ1(sex+t), φ2(sex), p(.) | 399.1635 | 7.8112 | 0.00503 | 0.0201 | 12 | 123.0082 |
| φ1(sex+t), φ2(.), p(sex) | 399.2942 | 7.9419 | 0.00472 | 0.0189 | 12 | 123.1388 |
| φ(t). p(t+sex) | 399.3786 | 8.0263 | 0.00452 | 0.0181 | 14 | 118.7645 |
| φ(t+sex). p(t) | 399.668 | 8.3157 | 0.00391 | 0.0156 | 14 | 119.0539 |
| φ1(sex*t), φ2(sex), p(t) | 399.8632 | 8.5109 | 0.00355 | 0.0142 | 21 | 103.0431 |
| φ(.). p(.) | 399.9249 | 8.5726 | 0.00344 | 0.0138 | 2 | 145.0061 |
| φ1(t), φ2(sex), p(sex) | 400.0126 | 8.6603 | 0.00329 | 0.0132 | 12 | 123.8572 |
| φ1(sex*t), φ2(.), p(sex) | 400.7149 | 9.3626 | 0.00232 | 0.0093 | 17 | 113.2721 |
| φ1(sex*t), φ2(sex), p(.) | 400.7906 | 9.4383 | 0.00223 | 0.0089 | 17 | 113.3477 |
| φ(sex). p(.) | 400.9143 | 9.562 | 0.0021 | 0.0084 | 3 | 143.9476 |
| φ(t+sex). p(t+sex) | 401.1829 | 9.8306 | 0.00183 | 0.0073 | 15 | 118.3115 |
| φ(.). p(sex) | 401.2975 | 9.9452 | 0.00173 | 0.0069 | 3 | 144.3309 |
| φ1(sex+t), φ2(sex), p(sex) | 401.3113 | 9.959 | 0.00172 | 0.0069 | 13 | 122.9357 |
| φ1(.), φ2(.), p(.) | 401.9158 | 10.5635 | 0.00127 | 0.0051 | 3 | 144.9492 |
| φ(sex). p(sex) | 402.7573 | 11.405 | 0.00083 | 0.0033 | 4 | 143.7266 |
| φ1(sex*t), φ2(sex), p(sex) | 402.8585 | 11.5062 | 0.00079 | 0.0032 | 18 | 113.101 |
| φ1(sex), φ2(.), p(.) | 402.8756 | 11.5233 | 0.00079 | 0.0032 | 4 | 143.8449 |
| φ1(.), φ2(.), p(sex) | 403.2174 | 11.8651 | 0.00066 | 0.0026 | 4 | 144.1866 |
| φ1(.), φ2(sex), p(.) | 403.6446 | 12.2923 | 0.00054 | 0.0022 | 4 | 144.6138 |
| φ1(sex), φ2(.), p(sex) | 404.7332 | 13.3809 | 0.00031 | 0.0012 | 5 | 143.6218 |
| φ1(.), φ2(t), p(t) | 404.8585 | 13.5062 | 0.00029 | 0.0012 | 14 | 124.2444 |
| φ1(.), φ2(sex), p(sex) | 405.0887 | 13.7364 | 0.00026 | 0.001 | 5 | 143.9773 |
| φ1(sex), φ2(t), p(t) | 406.1882 | 14.8359 | 0.00015 | 0.0006 | 15 | 123.3168 |
| φ1(.), φ2(sex+t), p(t) | 406.7921 | 15.4398 | 0.00011 | 0.0004 | 15 | 123.9207 |
| φ(t*sex). p(.) | 408.4963 | 17.144 | 0.00005 | 0.0002 | 17 | 121.0534 |
| φ(t*sex). p(sex) | 410.3277 | 18.9754 | 0.00002 | 0.0001 | 18 | 120.5702 |
| φ1(.), φ2(t), p(.) | 410.5303 | 19.178 | 0.00002 | 0.0001 | 10 | 138.761 |
| φ1(sex), φ2(t), p(.) | 411.5805 | 20.2282 | 0.00001 | 0 | 11 | 137.6271 |
| φ1(.), φ2(t), p(sex) | 412.132 | 20.7797 | 0.00001 | 0 | 11 | 138.1786 |
| φ(t*sex). p(t) | 412.2676 | 20.9153 | 0.00001 | 0 | 21 | 115.4474 |
| φ1(.), φ2(sex+t), p(.) | 412.4198 | 21.0675 | 0.00001 | 0 | 11 | 138.4664 |
| φ1(sex), φ2(t), p(sex) | 413.6533 | 22.301 | 0 | 0 | 12 | 137.498 |
| φ1(.), φ2(sex*t), p(t) | 413.9261 | 22.5738 | 0 | 0 | 22 | 114.7113 |
| φ1(.), φ2(sex+t), p(sex) | 414.1508 | 22.7985 | 0 | 0 | 12 | 137.9954 |
| φ1(.), φ2(sex*t), p(.) | 418.6674 | 27.3151 | 0 | 0 | 18 | 128.9099 |
| φ1(sex), φ2(sex-t), p(sex*t) | 420.1313 | 28.779 | 0 | 0 | 28 | 106.1057 |
| φ1(.), φ2(sex*t), p(sex) | 420.2175 | 28.8652 | 0 | 0 | 19 | 128.1257 |
